# Structure–property relationships and third-order nonlinearities in diketopyrrolopyrrole based D–π–A–π–D molecules

**DOI:** 10.3762/bjoc.13.235

**Published:** 2017-11-08

**Authors:** Jan Podlesný, Lenka Dokládalová, Oldřich Pytela, Adam Urbanec, Milan Klikar, Numan Almonasy, Tomáš Mikysek, Jaroslav Jedryka, Iwan V Kityk, Filip Bureš

**Affiliations:** 1 Institute of Organic Chemistry and Technology, Faculty of Chemical Technology, University of Pardubice, Studentská 573, Pardubice, 53210, Czech Republic; 2 Centre of Organic Chemistry Ltd., Rybitví 296, Rybitví, 53354, Czech Republic; 3 Department of Analytical Chemistry, Faculty of Chemical Technology, University of Pardubice, Studentská 573, Pardubice, 53210, Czech Republic,; 4 Institute of Optoelectronics and Measuring Systems, Faculty of Electrical Engineering, Czestochowa University of Technology, Armii Krajowej 17, Czestochowa, 42-200, Poland

**Keywords:** calculations, diketopyrrolopyrrole, electrochemistry, electronic spectra, push–pull, third-harmonic generation

## Abstract

Nine new quadrupolar chromophores based on diketopyrrolopyrrole were designed and prepared by cross-coupling reactions. The property tuning has been achieved by structural variation of the peripheral substituents (donor) and enlargement of the π-system. Fundamental properties of target molecules were studied by differential scanning calorimetry, electrochemistry, and absorption and emission spectra. Nonlinear optical properties were studied by measuring the third harmonic generation. The experimental data were completed by quantum-chemical calculations and structure–property relationships were elucidated.

## Introduction

Known for more than 40 years, diketopyrrolopyrroles (DPP) represent an unique class of organic molecules based on central, fused, and conjugated bicyclic lactams of 2,5-dihydropyrrolo[3,4-*c*]pyrrole-1,4-dione. After its serendipitous discovery by Farnum et al. in 1974 [1] and subsequent first applications as organic, insoluble, and high-performance pigments [2], DPPs have significantly infiltrated organic electronics as functional dyes. The number of recently appeared review articles [3–8] clearly demonstrates their wide application potential, which spans organic solar cells (OSC), organic field-effect transistors (OFET), organic light-emitting diodes (OLED), fluorophores, probes for ion sensing, functional polymers, and more recently also chromophores with nonlinear optical (NLO) activity. The latter property is mostly dictated by their conjugated character, electron-accepting behavior of the DPP scaffold, strong emissive character, and eventually intramolecular charge-transfer (ICT) [9–10] from the peripheral donors to the central DPP acceptor. Due to their generally D–π–A–π–D character and thus resulting centrosymmetric arrangement, the DPP derivatives were most frequently studied as third-order optical NLOphores, in particular as two-photon absorbers (2PA). In this respect, the central DPP bicyclic lactam is often decorated with electron donors such as alkoxy- or dialkylamino groups [11–12], triphenylamine [13–14], heterocyclic carbazole [15], thiophene [16–17], furan [18], and organometallic ferrocene [19]. 2PA-active DPPs were utilized in two-photon excited fluorescence microscopy and bioimaging [13–14], most often upon their further structural tuning towards higher polar character and solubility in water [20–22]. Their linking to porphyrins proved to be a useful strategy for two-photon photodynamic therapy [23–25]. Moreover, some DPP derivatives also showed aggregation induced emission (AIE) [13–14] and the ability to selectively sense fluoride ions [26–28]. The modern era of DPP chromophores has been opened by Gryko et al. [3,11–12,18,20–21,29] who have demonstrated their large and tunable two-photon absorption cross-section (δ_2PA_) generally ranging from 100–2500 GM, but for instance DPPs end-capped with imidazolium [21] or dendritic thiophenes [16] showed δ_2PA_ of 4000 and 7000 GM, respectively. However, to the best of our knowledge, the third-harmonic generation (THG) NLO process has not been investigated for DPP derivatives. The most common applications of organic molecules with THG activity are directed towards all-optical signal processing and optical imaging [30–31].

**Figure 1:**
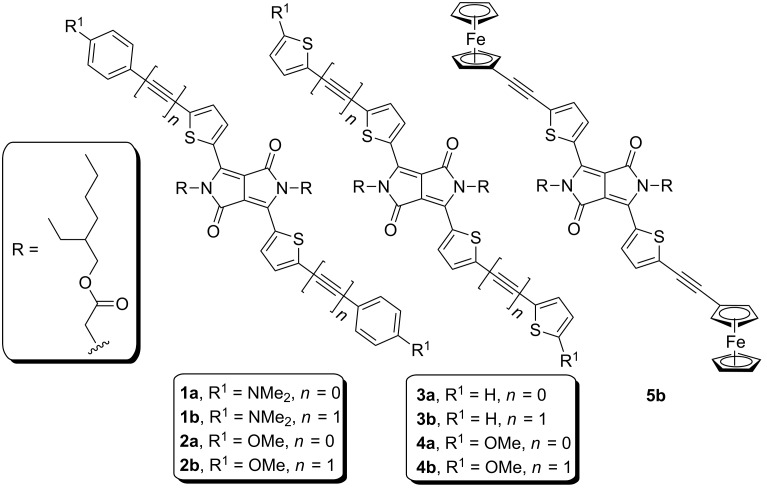
General structure of investigated DPP derivatives **1**–**5**.

Hence, as a continuation of our research activity on incorporating heteroaromatic moieties in push–pull molecules [9–10], we report herein the systematic modification of the 3,6-dithienyl-2,5-dihydropyrrolo[3,4-*c*]pyrrole-1,4-dione central acceptor with *N*,*N*-dimethylanilino (DMA), methoxyphenyl, thiophene, methoxythiophene, and ferrocene peripheral electron donors (Figure 1). Fundamental properties of D–π–A–π–D chromophores **1**–**5** were further investigated by differential scanning calorimetry (DSC), electrochemistry, absorption and emission spectra, DFT calculations, and THG measurements.

## Results and Discussion

### Synthesis of DPP derivatives **1**–**5**

**Scheme 1:**
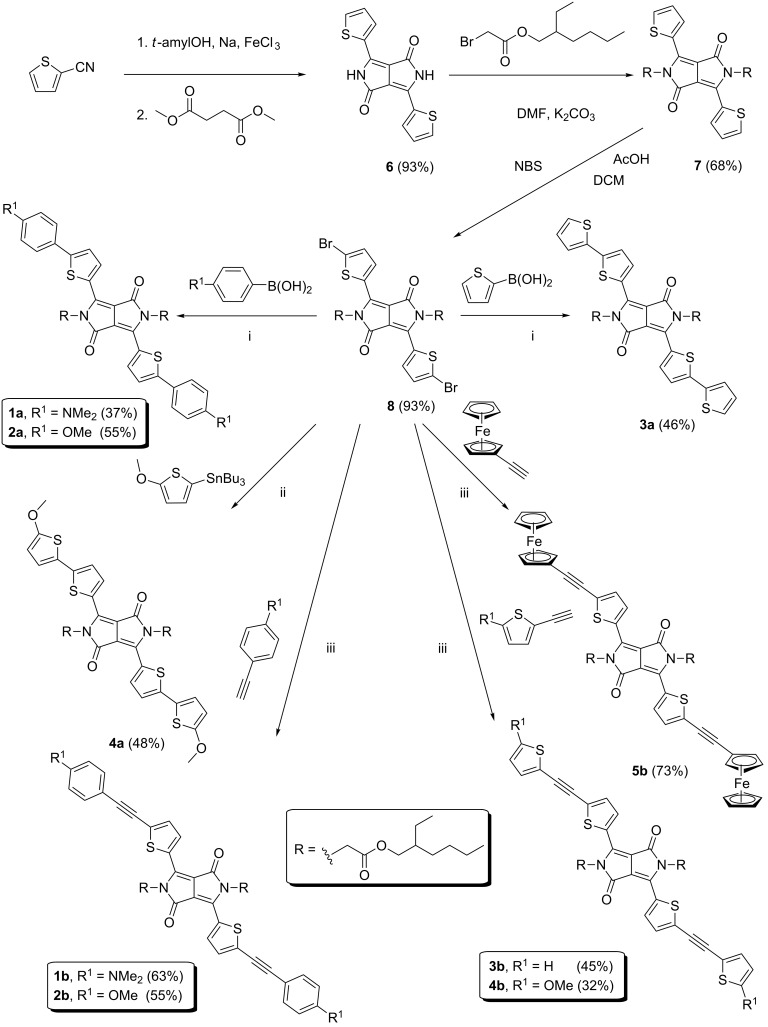
Synthesis of target DPP chromophores **1**–**5**. (i) PdCl_2_(PPh_3_)_2_, Na_2_CO_3_, THF, H_2_O; (ii) PdCl_2_(PPh_3_)_2_, THF; (iii) PdCl_2_(PPh_3_)_2_, CuI, TEA, THF.

According to the π-linker structure, two series of target chromophores **a** and **b** can be distinguished (*n* = 0 or 1). Whereas chromophores in series **a** possess the donor directly connected to the DPP acceptor, in series **b** are these moieties separated by an additional acetylene unit. The reaction sequence leading to target chromophores **1**–**5** consists of a three-step preparation of dibromo derivative **8** and its final cross-coupling reactions (Scheme 1). The construction of the DPP central scaffold **6** was accomplished by a well-known reaction between thiophene-2-carbonitrile and dimethyl succinate in the presence of sodium *tert*-amylalcoholate generated in situ [32]. The reaction provided **6** with a high yield of 93%. The dark red pigment **6** is only sparingly soluble in commonly used solvents, and its purification was carried out by multiple washing with methanol. However, its twofold *N*-alkylation using 2-ethylhexyl bromoacetate in DMF/K_2_CO_3_ afforded **7** with a satisfactory yield of 68% and with significantly increased solubility [32]. It should be noted that other alkylating reagents, bases, and solvents provided the desired products with much lower yield and purity. Subsequent treatment of **7** with *N*-bromosuccinimide (NBS) smoothly afforded dibromo derivative **8** with a high yield of 93%. Bromination of **7** using a larger excess of NBS resulted in its decomposition. According to the current literature reports [18–19,27], DPP derivative **8** undergoes smooth cross-coupling reactions. Hence, we have utilized twofold Suzuki–Miyaura and Migita–Stille reactions leading to chromophores **1a**–**3a** and **4a**, respectively. Commercially available 4-(*N*,*N*-dimethylamino)phenylboronic, 4-methoxyphenylboronic, and 2-thienylboronic acids completed with tributyl(5-methoxythiophen-2-yl)stannane, prepared from 2-methoxythiophene [33], were used.

Chromophore **4a** was also attempted by Suzuki–Miyaura reaction using the corresponding 5-methoxythiophen-2-ylboronic acid pinacol ester [34], however, this reaction proved to be very sluggish. A similar reaction with ferroceneboronic acid provided no product and, therefore, compound **5a** was not prepared. This was most likely due to the instability and the low reactivity of these two boron derivatives [34–35]. Twofold Sonogashira cross-coupling was utilized towards chromophores **1b**–**5b**. All cross-coupling reactions were carried out in THF with PdCl_2_(PPh_3_)_2_ as palladium precatalyst. The reaction time was approximately 24 hours to achieve sufficient conversion (32–73%). Despite bearing two large alkyl groups, the solubility of the target chromophores is relatively low, which partially complicated their purification. Hence, column chromatography with very slow elution followed by subsequent crystallization has been necessary. Only derivative **5b** was sufficiently pure after column chromatography without a need of further crystallization. Hence, this ferrocene derivative was prepared with the highest yield of 73%. In the solid state, all target chromophores resemble dark metallic solids.

### Differential scanning calorimetry

**Figure 2:**
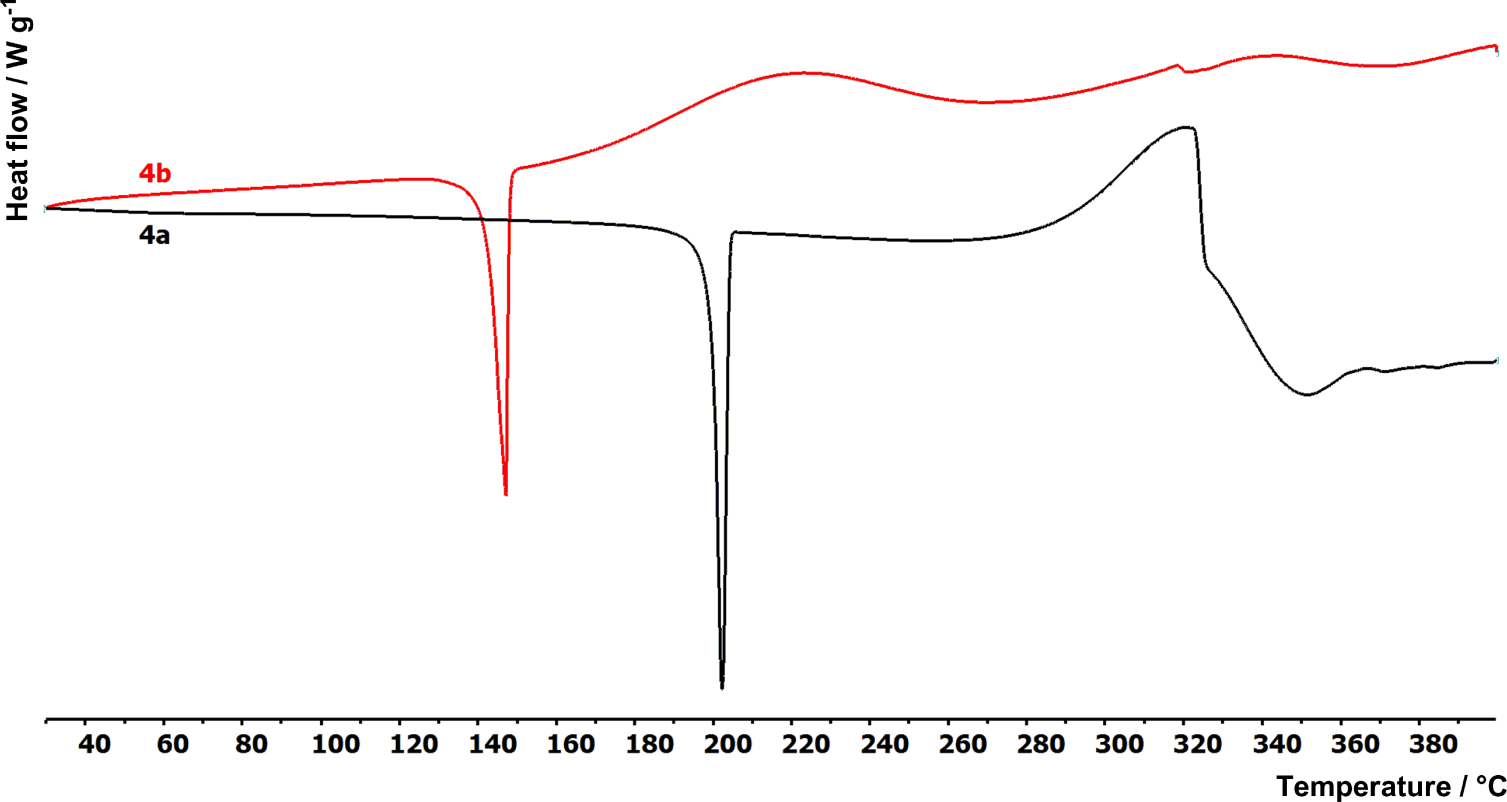
Thermograms of representative chromophores **4a** and **4b**.

The thermal behaviour of compounds **1**–**5b** was studied by differential scanning calorimetry (DSC). Figure 2 shows the thermograms of the representative compounds **4a** and **4b** while Table 1 lists the measured melting temperatures (*T*_m_) and temperatures of thermal decomposition (*T*_d_). The measured melting points of derivatives in series **a** and **b** range from 200 to 261 °C and from 142 to 215 °C, respectively. All compounds provided a very sharp peak of melting while the peaks of the decomposition were mostly broad (50–80 °C). The following thermal structure–property relationships can be concluded from the measured data:

- Compounds in series **a** are stable even in the liquid phase and decompose far beyond their melting points (e.g., **2a** with *T*_m_ = 244 °C and *T*_d_ = 345 °C).
- Compounds in series **b** bearing an additional acetylene linker decompose almost immediately after their melting (e.g., **2b** with *T*_m_ = 175 °C and *T*_d_ = 189 °C).
- In pairs of chromophores, the substance without triple bond has an approximately 50 °C higher melting point (e.g., **1a**/**1b** with *T*_m_ = 261/215 °C).
- Thus, the insertion of an acetylene linker decreases the melting point and causes thermal instability in the liquid phase.
- Derivatives **1a**/**2a** containing a 1,4-phenylene linker showed the highest *T*_m_ and *T*_d_ values of 261/244 and 330/345 °C, respectively.
- The 2,5-thienylene linker decreased the melting point approximately by 40 °C (e.g., **2a**/**4a** with *T*_m_ = 244/200 °C).
- End-capping the DPP derivative with a ferrocene donor brings improved thermal stability (e.g., compound **5b** with the second highest *T*_m_ and *T*_d_ values within the series **b**).

**Table 1:** Thermal, electrochemical, and DFT calculated data for chromophores **1**–**5**.

Com.	*T*_m_ [°C]^a^	*T*_d_ [°C]^a^	*E*_1/2(ox1)_ [V]^b^	*E*_1/2(red1)_ [V]^b^	*E*_HOMO_ [eV]^c^	*E*_LUMO_ [eV]^c^	Δ*E* [eV]	*E*_HOMO_^DFT^ [eV]^d^	*E*_LUMO_^DFT^ [eV]^d^	Δ*E*^DFT^ [eV]

**1a**	261	330	0.62	−1.03	−4.97	−3.32	1.65	−4.92	−2.93	1.99
**2a**	244	345	0.86	−0.84	−5.30	−3.40	1.90	−5.24	−3.05	2.20
**3a**	206	329	0.95	−0.95	−5.42	−3.50	1.92	−5.30	−3.15	2.15
**4a**	200	289	1.11	−0.80	−5.16	−3.43	1.73	−5.07	−3.04	2.03
**1b**	215	227	1.07	−0.85	−5.21	−3.52	1.70	−5.04	−3.15	1.89
**2b**	175	189	1.11	−0.75	−5.46	−3.55	1.91	−5.31	−3.24	2.07
**3b**	158	173	0.81	−0.92	−5.46	−3.60	1.86	−5.27	−3.21	2.06
**4b**	142	170	1.08	−0.75	−5.43	−3.60	1.82	−5.20	−3.23	1.97
**5b**	188	192	0.69	−0.78	−5.04	−3.57	1.47	−5.31	−3.21	2.10

^a^Determined by DSC in open aluminous crucibles under N_2_ inert atmosphere and with a scanning rate of 3 °C/min within the range of 25–450 °C. Melting point and temperature of decomposition were determined as intersection of the baseline and tangent of the peak (onset point). ^b^*E*_1/2(ox1)_ and *E*_1/2(red1)_ are half-wave potentials of the first oxidation and reduction measured in DMF; all potentials are given vs SCE. ^c^Recalculated from the *E*_1/2(ox1/red1)_ according the equation –*E*_HOMO/LUMO_ = *E*_1/2(ox1/red1)_ + 4.35 ([36]). ^d^Calculated at the DFT B3LYP/6-311++G(2df,p) level.

### Electrochemistry

Electrochemical measurements of all target compounds were carried out in *N,N*-dimethylformamide containing 0.1 M Bu_4_NPF_6_ in a three electrode cell by cyclic voltammetry (CV) and rotating disk voltammetry (RDV). The working electrode was a platinum disk (2 mm in diameter) for CV and RDV experiments. As the reference and auxiliary electrodes were used saturated calomel electrodes (SCE) separated by a bridge filled with supporting electrolyte and Pt wire, respectively. All potentials are given vs SCE. Voltammetric measurements were performed using a potentiostat PGSTAT 128N (AUTOLAB, Metrohm Autolab B.V., Utrecht, The Netherlands) operated via NOVA 1.11 software.

**Figure 3:**
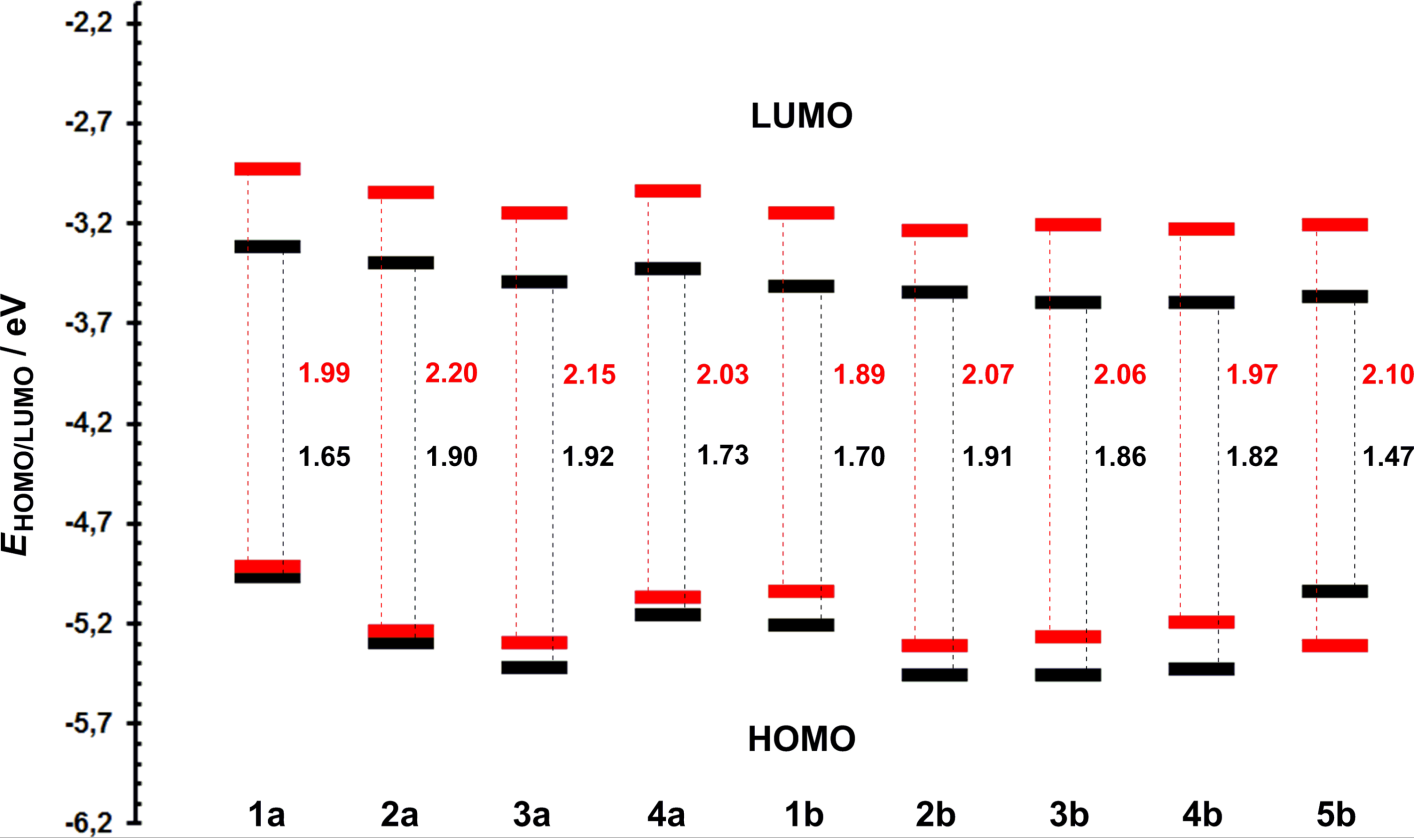
Energy level diagram of the electrochemical (black) and DFT (red) derived energies of the *E*_HOMO/LUMO_ for chromophores **1**–**5**.

The reduction for all compounds is represented by reversible one-electron process with a peak separation about 70 mV. On the other hand, only compounds **1a**,**b**, **4a**, and **5b** showed reversible oxidations, the others represent an irreversible process even at higher scan rates (e.g., 1 V/s). Representative CV curves can be found in the Supporting Information File 1. Table 1 lists the measured half-wave potentials of the first oxidation (*E*_1/2(ox1)_) and reduction (*E*_1/2(red1)_), which were further recalculated to the energies of the HOMO (*E*_HOMO_) and LUMO (*E*_LUMO_) [36]. These values and their differences (Δ*E*) are also visualized in the energy level diagram shown in Figure 3. As can be seen, both energy levels are a function of the DPP substitution. Whereas the *E*_LUMO_ is relatively steady (*E*_LUMO_ = −3.32 to −3.36 eV), the principal changes are seen in the *E*_HOMO_, which ranges from −4.97 to −5.46 eV. Hence, the electrochemical gaps (Δ*E*) were found within the range of 1.47 to 1.92 eV. From the electrochemical data we can deduce the following structure–property relationships:

- Increasing the electron-donating character of the peripheral substituent shifts the *E*_HOMO_ to more positive values, slightly deepened *E*_LUMO_, and reduces the Δ*E* within the order of **1** (dimethylanilino) > **2** (4-methoxyphenyl) > **4** (5-methoxythiophen-2-yl) > **3** (thiophen-2-yl).
- The organometallic ferrocene in **5b** caused reduction of the HOMO–LUMO gap up to 1.47 eV and proved to be the strongest electron donor within the studied series of compounds. This is in accordance with our previous observations [9,35].
- Except for chromophores **3a**,**b**, insertion of an additional acetylene linker rather extended the electrochemical HOMO–LUMO gap (series **a** vs **b**).

### Linear optical properties

**Figure 4:**
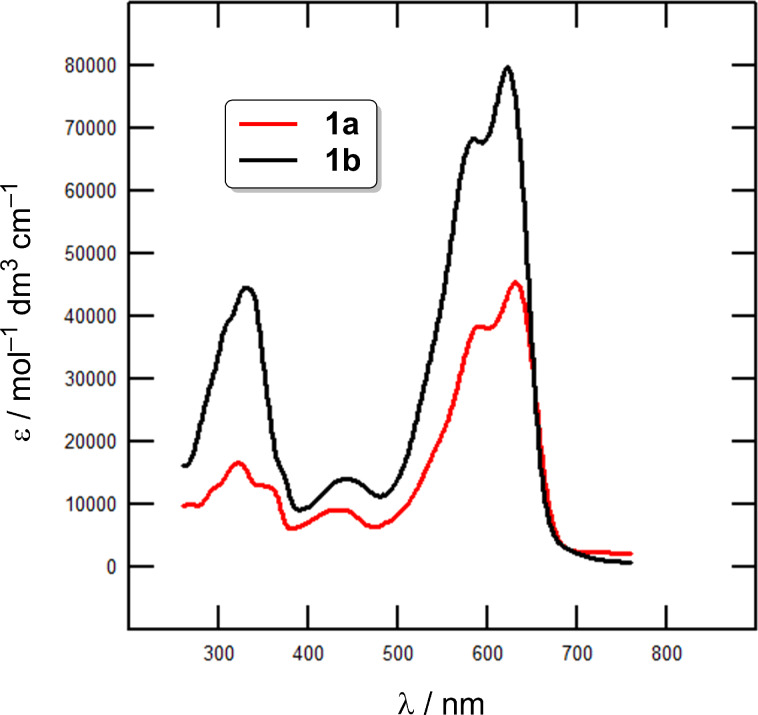
UV–vis absorption spectra of chromophores **1a** and **1b** in 1,4-dioxane at a concentration of 1 × 10^−5^ M.

**Figure 5:**
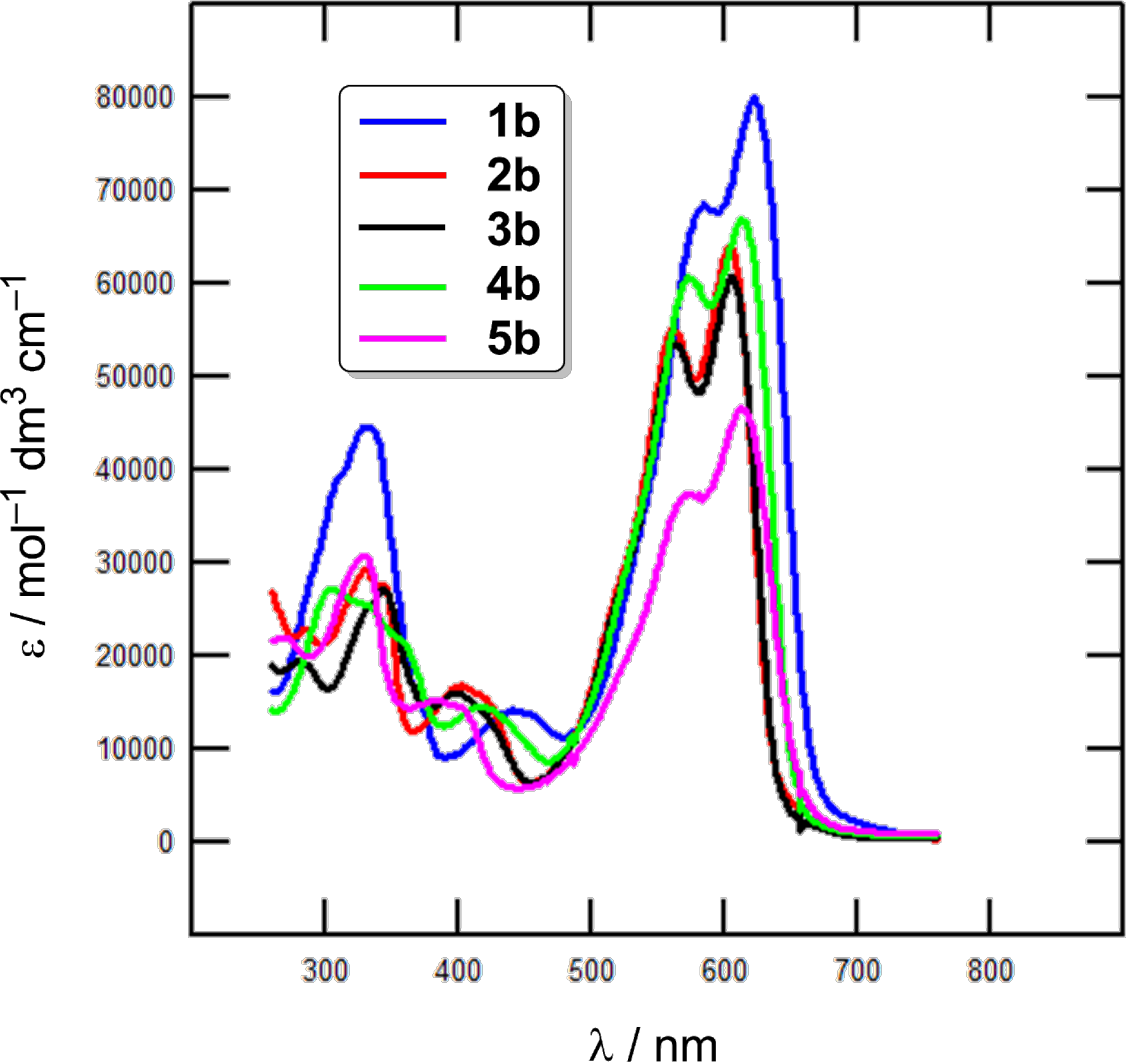
UV–vis absorption spectra of chromophores **1b**–**5b** in 1,4-dioxane at a concentration of 1 × 10^−5^ M.

Absorption spectra of target chromophores were measured in 1,4-dioxane at a concentration of 1 × 10^−5^ M. Representative spectra of chromophores in series **a** and **b** are shown in Figure 4 and Figure 5 as a dependence of the molar absorption coefficient (ε) on the wavelength (λ). Table 2 summarizes the measured longest-wavelength absorption/emission maxima (λ_max_^A/F^), terminal absorptions (λ_end_), Stokes shifts, and quantum yields of fluorescence (q^F^). The absorption spectra consist of two intense low-energy charge-transfer bands located within the range of 550–650 nm accompanied by weak high-energy bands found within 300–450 nm. This is a typical feature of DPP-based D–π–A–π–D chromophores [12,18–19]. The two spectra shown in Figure 4 demonstrate the impact of the π-system extension by an acetylene linker. When going from **1a** to **1b**, the spectrum shifts slightly hypsochromically (Δλ_max_^A^ = 10 nm) and the optical gap was extended from 1.96 to 1.99 eV. Despite its extending effect, the acetylene π-linker also shows a low rotational barrier (≈0.025 eV) and various dynamic conformations may exist in series **b**, which lowers the D–A interaction and results in hypsochromically shifted spectra [37–38]. This also corresponds with the aforementioned electrochemical observations. However, the chromophores in series **b** with two additional triple bonds possess generally higher molar absorption coefficients as a result of the π-system enlargement. Figure 5 demonstrates the impact of the peripheral electron donors appended to the DPP core, namely in well-evaluated series **b**. For instance, a replacement of *N*,*N*-dimethylamino groups in **1b** (λ_max_^A^ = 622 nm) by two methoxy groups as in **2b** (λ_max_^A^ = 604 nm) resulted in a hypsochromic shift of 18 nm. An even more pronounced blue shift can be seen in series **a**, e.g., **1a** vs **2a** (Δλ_max_^A^ = 32 nm). However, chromophore **2b** with a 4-methoxyphenyl donor possesses almost identical absorption maxima as thiophen-2-yl derivative **3b** (λ_max_^A^ = 605 nm), implying their similar electron-releasing abilities. A combination of both methoxy and thiophene auxiliary donors as in **4b** shifted its absorption maxima to 613 nm. Ferrocene-terminated DPP derivative **5b** showed an absorption maximum at 614 nm. The most bathochromically shifted λ_max_^A^ were measured for chromophores **1a**/**4a** and **1b**/**4b**/**5b** featuring *N*,*N*-dimethylanilino, 5-methoxythiophen-2-yl or ferrocene donors.

Emission spectra of all prepared DPP derivatives were measured in 1,4-dioxane with excitation under wavelength corresponding to the absorption maxima (Table 2, see also Supporting Information File 1). All examined compounds except **5b** showed fluorescent behavior. The measured fluorescence spectra show a vibrational structure and are mirror images to the first absorption bands. The positions of the emission maxima copy trends seen in the absorption spectra. Increasing the donating ability of the attached peripheral R^1^-substituents results in a slight bathochromic shift, whereas extension of the π-system by acetylene linkers has an opposite effect. The measured quantum yields of fluorescence are within the range of 0.17–0.35 with no direct trends. However, a slight decrease of q^F^ can be found when going from the chromophores bearing weaker donors (e.g., **2a**/**b**) to chromophores with strong donors such as NMe_2_ or ferrocene (e.g., **1a**/**b** or **5b**).

**Table 2:** Optical properties of chromophores **1**–**5**.

Com.	λ_max_^A^ [nm (eV)]^a^	ε [M^−1^·cm^−1^]^a^	λ_end_^A^ [nm (eV)]^b^	λ_max_^F^ [nm (eV)]^c^	Stokes shift [cm^−1^ (eV)]	q^Fc^	THG^d^ [pm^2^/V^2^]	γ^e^ [×10^−24^ esu]

**1a**	632 (1.96)	45475	676 (1.83)	672 (1.85)	942 (0.11)	0.20	102	1.81
**2a**	600 (2.07)	44228	633 (1.96)	623 (1.99)	615 (0.08)	0.35	115	1.22
**3a**	604 (2.05)	50621	641 (1.93)	633 (1.96)	759 (0.09)	0.17	67	11.55
**4a**	623 (1.99)	61744	663 (1.87)	655 (1.89)	758 (0.10)	0.21	67	5.26
**1b**	622 (1.99)	79982	665 (1.86)	657 (1.89)	856 (0.10)	0.19	130	3.94
**2b**	604 (2.05)	63970	638 (1.94)	627 (1.98)	607 (0.07)	0.28	113	2.46
**3b**	605 (2.05)	60613	640 (1.94)	629 (1.97)	631 (0.08)	0.24	45	36.06
**4b**	613 (2.02)	66833	653 (1.90)	643 (1.93)	761 (0.09)	0.18	108	13.19
**5b**	614 (2.02)	46626	657 (1.89)	–	–	–	15	–

**^a^**Measured in 1,4-dioxane at a concentration of 1 × 10^−5^ M. ^b^Intersection of the low-energy edge of the CT-band (linear regression) with the horizontal axis. ^c^Measured as diluted samples in 1,4-dioxane, (4-(dicyanomethylene)-2-methyl-6-(4-dimethylaminostyryl)-4*H*-pyran in *n*-propanol was used as a fluorescent standard for determining the fluorescence quantum yields (λ_max_^F^ = 614 nm, q^F^ = 0.57). ^d^Measured with a 1064 nm source fundamental laser beam in reflected light geometry. ^e^Calculated by PM7 semi-empirical method from the DFT-optimized geometries (except for **5b**).

### Nonlinear optical properties

The third harmonic generation (THG) measurements of target chromophores **1**–**5** were carried out by using a 1064 nm Nd:YAG source beam. Due to the relatively high absorption of chromophores **1**–**5** around 355 nm, the observation of the THG was possible only in the reflected light geometry. However, the absorption bands found within this spectral range are up to four-times smaller than around λ_max_^A^ (Figure 4 and Figure 5). Details of the experimental setup are given in Supporting Information File 2.

**Figure 6:**
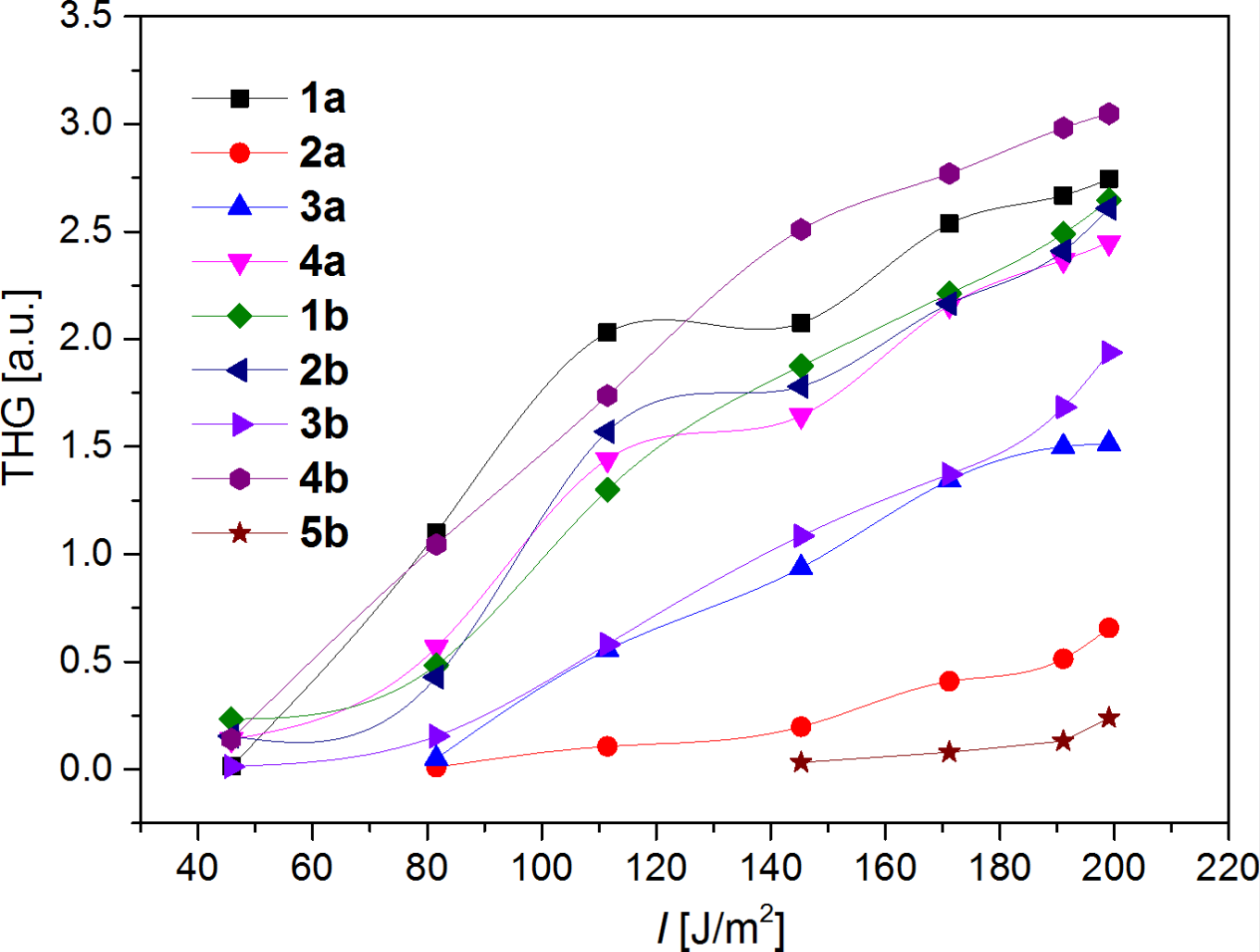
Typical THG dependences vs the fundamental energy density.

The effective contribution to the THG has been measured on a thin layer of **1**–**5** (up to 70 nm), which further favor enhanced third order optical effects [39]. Figure 6 shows the dependences of the output reflected THG versus the fundamental energy density. The absolute values of the THG are given in Table 2. The experimental THG values showed some weak trends, which can be summarized as follows:

- Generally largest THG responses were recorded for chromophores with extended π-system in series **b**.
- Chromophore **1b** bearing a strong *N*,*N*-dimethylamino donor and an extended π-system showed the highest third-order NLO response of 130 pm^2^/V^2^.
- However, a comparison of electron donors is less straightforward. Whereas in series **b** chromophore **1b** showed larger THG than **2b**, the situation is completely opposite in series **a**.
- Attaching ferrocene donors as in **5b** has a very detrimental effect on the NLO response.
- Despite the THG efficiency usually increases with narrowing HOMO–LUMO gap, in the present series of DPP molecules is this trend less observable.
- THG responses of DPP chromophores **1**–**5** are affected by their strong linear absorption nearby the third harmonic generation (≈355 nm) and occurrence of thin nanolayers for the THG wavelength.
- The electronic hyperpolarizabilities, which are mainly defined by the dipole moments and vectorial differences between the excited and ground state moments, are not dominant contributions in **1**–**5**.

In order to place the obtained THG values of DPP derivatives **1**–**5** in a broader context, we compare herein these data with some earlier studied materials. For instance, *p*-*N*,*N*’-dimethylaniline tetrathiafulvalene derivatives showed third-order nonlinear optical susceptibilities within a range of 20–32 pm^2^/V^2^ [40], whereas quinoline push-pull derivatives embedded in polymethylmethacrylate matrix possess a THG susceptibility equal to 46 pm^2^/V^2^ [41]. Reference inorganic single crystals of α-BiB_3_O_6_ provided under the same experimental conditions a THG response of 105 pm^2^/V^2^ [42]. Hence, the measured THG values of 15–130 pm^2^/V^2^ for DPP derivatives **1**–**5** are comparable with the know data, some of them even exceeded the reference inorganic material and, therefore, DPP derivatives can be considered as promising organic materials with tunable third-order nonlinear optical properties.

### DFT calculations

Spatial and electronic properties of all target chromophores **1**–**5** were investigated at the DFT level by using the Gaussian W09 package [43]. The geometries of molecules **1**–**5** were optimized by using the DFT B3LYP/6-311G(2df,p) method. Energies of the HOMO and the LUMO, their differences and ground-state dipole moments μ were calculated on the DFT B3LYP/6-311++G(2df,p) level (Table 1). Second hyperpolarizabilities were calculated by using the PM7 semi-empirical method implemented in MOPAC [44] and DFT-optimized geometries (Table 2).

**Figure 7:**
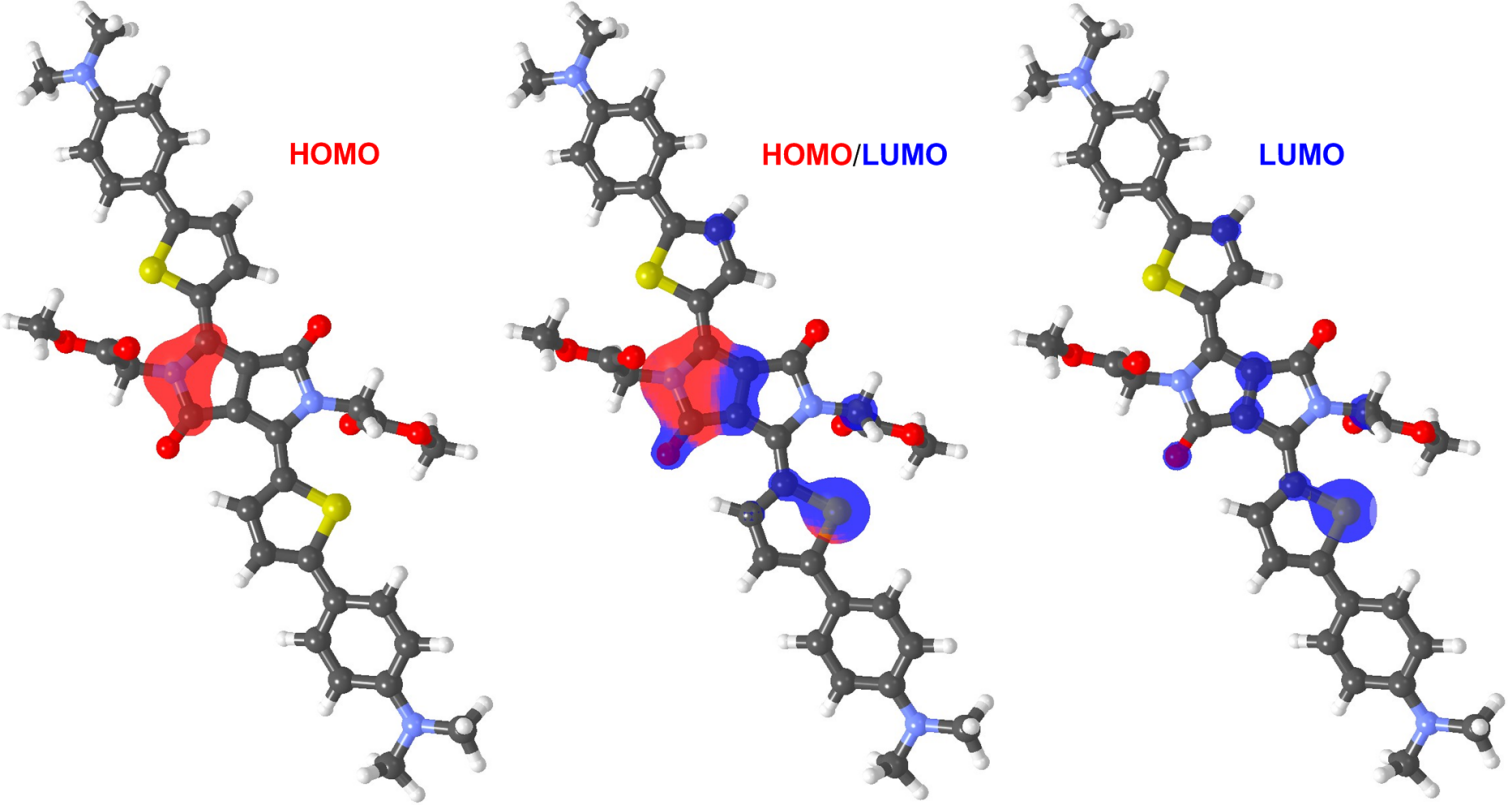
HOMO (red) and LUMO (blue) localizations in **1a** (ethylhexyl chains truncated).

The calculated energies of the HOMOs and the LUMOs of compounds **1**–**5** range from −5.31 to −4.92 eV and from −3.24 to −2.93 eV, respectively (Table 1). They are a function of the attached peripheral donor substituent and type of the π-linker. As can be seen from the energy level diagram (Figure 3), the calculated and electrochemically derived HOMO and LUMO levels are in a good agreement and, therefore, the used DFT method is capable to describe trends in the given series of molecules. Similarly to electrochemical measurements, DFT calculations indicated almost steady LUMO, the principal changes are seen in the HOMO level. This reflects structural variations in **1**–**5** made exclusively in the donor part. The HOMO and LUMO localizations in representative chromophore **1a** are shown in Figure 7, for complete listing see Supporting Information File 1. In general, both the LUMO and the HOMO are predominantly localized on the central DPP core and appended thiophene rings, with relatively weak charge separation. This implies that D–A interaction from the peripheral donors to the central DPP acceptor is relatively weak, which is also reflected by small changes seen in the aforementioned electrochemical and spectral data upon structural tuning. DFT calculations also revealed that all target chromophores **1**–**5** are centrosymmetric (*C*_i_ point group) and, therefore, they possess zero ground state dipole moments. The PM7-calculated second hyperpolarizabilities γ were capable to distinguish π-system extension (generally higher NLO response for chromophores in series **b**) as well as electron-releasing power of the particular donors (e.g., **1a** vs **2a** or **1b** vs **2b**). As can be seen, the PM7 calculations strongly overestimate thiophene-derived chromophores (e.g., **3a**/**4a** and **3b**/**4b**) and are not capable to properly calculate organometallic ferrocene derivatives **5b**. However, this is its common feature [45–46].

## Conclusion

In conclusion, we have designed a series of diketopyrrolopyrrole derivatives having D–π–A–π–D arrangement. Nine new chromophores were conveniently and in modular way prepared by Suzuki–Miyaura, Migita–Stille, and Sonogashira cross-coupling reactions. The chromophores differ in the π-system extension as well as in the peripheral substitution. The thermal stability of **1**–**5** is mostly affected by the presence of acetylene linkers, 1,4-phenylene and 2,5-thienylene linkers, and ferrocene termini. From the electrochemical data we can conclude that variation of the peripheral donors influences mostly the HOMO, which dictates the HOMO–LUMO gap. Absorption spectra showed two bands localized around 600 nm, which position weakly depend on the peripheral substituents as well as the π-system extension/composition. The measured THG responses were found within the range of 15 to 130 pm^2^/V^2^ and are mostly affected by the π-system enlargement and less by the peripheral donors.

## Supporting Information

File 1 Experimental procedures and characterization of compounds, THG measurements.

File 2 ^1^H and ^13^C NMR spectra, HR-MALDI-MS spectra, CV curves, UV–vis absorption/emission spectra, and HOMO/LUMO localizations.
