# Supplementary material for: Structure–property relationships and third-order nonlinearities in diketopyrrolopyrrole based D–π–A–π–D molecules
Source: Beilstein J Org Chem. 2017 Nov 8;13:2374–84. doi: 10.3762/bjoc.13.235 (PMC5687008; doi:10.3762/bjoc.13.235)
Supplement: File 1 — Experimental procedures and characterization of compounds, THG measurements. [file Beilstein_J_Org_Chem-13-2374-s001.pdf]

Supporting Information File 1

for

Structure–property relationships and third-order nonlinearities in diketopyrrolopyrrole based D–π–A–π–D molecules

Jan Podlesný[1], Lenka Dokládalová[2], Oldřich Pytela[1], Adam Urbanec[1], Milan Klikar[1], Numan Almonasy[1], Tomáš Mikysek[3], Jaroslav Jedryka[4], Iwan V. Kityk[4] and Filip Bureš*[1]

Address: [1]Institute of Organic Chemistry and Technology, Faculty of Chemical Technology, University of Pardubice, Studentská 573, Pardubice, 53210, Czech Republic, [2]Centre of Organic Chemistry Ltd., Rybitví 296, Rybitví, 53354, Czech Republic, [3]Department of Analytical Chemistry, Faculty of Chemical Technology, University of Pardubice, Studentská 573, Pardubice, 53210, Czech Republic, and [4]Institute of Optoelectronics and Measuring Systems, Faculty of Electrical Engineering, Czestochowa University of Technology, Armii Krajowej 17, Czestochowa, 42-200, Poland

Email: Filip Bureš - filip.bures@upce.cz

* Corresponding author

Experimental procedures and characterization of compounds, THG measurements

Table of contents

Experimental procedures and characterization of compounds S3

3,6-Di(thiophen-2-yl)-2,5-dihydropyrrolo[3,4-*c*]pyrrol-1,4-dione (**6**) S5

N,N'-Dialkylated DPP derivative **7** S5

Dibromo DPP derivative **8** S6

General method for Suzuki-Miyaura cross-coupling reaction S7

Chromophore **1a** S7

Chromophore **2a** S8

Chromophore **3a** S8

Chromophore **4a** S9

General method for Sonogashira cross-coupling reaction S10

Chromophore **1b** S10

Chromophore **2b** S11

Chromophore **3b** S11

Chromophore **4b** S12

Chromophore **5b** S12

THG measurement's method S14

References S15

Experimental procedures and characterization of compounds

Reagents and solvents were reagent-grade and were purchased from Penta, Aldrich, and Fluka and used as received. Dry THF was freshly distilled from Na/K alloy and diphenylmethanone under inert argon atmosphere. Used solvents were evaporated on a Heidolph Laborta 4001 rotary evaporator. Cross-coupling reactions were carried out at vacuum-inert line in Schlenk's flasks. Column chromatography was carried out with silica gel 60 (particle size 0.040–0.063 mm, 230–400 mesh; Merck) and commercially available solvents. Thin-layer chromatography (TLC) was conducted on aluminum sheets coated with silica gel 60 F_{254} obtained from Merck, with visualization by UV lamp (254 or 360 nm). Melting points (mp) were measured on a Büchi B-540 melting-point apparatus in open capillaries and are uncorrected. ^{1}H and ^{13}C NMR spectra were recorded in $CDCl_3$ at 400/100 MHz with a Bruker AVANCE III or 500/125 MHz with Bruker Ascend^{TM} at 25 °C. Chemical shifts are reported in ppm relative to the signal of Me_4Si. The residual solvent signal in the ^{1}H and ^{13}C NMR spectra was used as an internal reference ($CDCl_3$ – 7.25 and 77.23 ppm). Coupling constants (*J*) are given in Hz. The apparent resonance multiplicity is described as s (singlet), d (doublet), t (triplet), q (quartet) and m (multiplet). Signal overlap of 2-ethylhexyl chain CH group with signal of residual water can be observed in ^{1}H NMR spectra. High-resolution MALDI mass spectra were measured using method "dried droplet" on a MALDI mass spectrometer LTQ Orbitrap XL (Thermo Fisher Scientific, Germany) equipped with nitrogen UV laser (337 nm, 60 Hz). The LTQ Orbitrap instrument was operated in positive ion mode over a normal mass range (*m*/*z* 50–2000) with the resolution 100.000 at *m*/*z* 400. The used matrix was 2,5-dihydroxybenzoic acid (DHB). IR spectra were recorded as neat using HATR adapter on a Perkin Elmer FTI Spectrum BX spectrometer. UV–vis spectra were

recorded on a HP 8453 spectrophotometer in 1,4-dioxane (*c* 1 × 10^{-5} mol/L). These solutions were diluted 10x for fluorescent measurement, absorbance A_{max} (0.06–0.1). Emission spectra were recorded on fluorescent spectrofotometer Perkin-Elmer LS-55. 4-(dicyanomethylen)-2-methyl-6-(4-dimethylaminostyryl)-4*H*-pyrane in *n*-propanol was used as fluorescent standard for determination of quantum yields of fluorescence (λ_{Fmax} = 614 nm, q_F = 0.57). Thermal properties were measured by differential scanning calorimetry DSC with a Mettler-Toledo STARe System DSC 2/700 equipped with FRS 6 ceramic sensor and cooling system HUBERT TC100-MT RC 23. DSC curves were determined in open aluminous crucibles under N_2 inert atmosphere and with a scanning rate of 3 °C/min within the range 25–450 °C. Melting point and temperature of decomposition were determined as intersection of baseline and tangent of peak (oneset point). Electrochemical measurements were carried out in *N*,*N*-dimethylformamide containing 0.1 M Bu_4NPF_6 in a three electrode cell by cyclic voltammetry (CV), rotating disk voltammetry (RDV). The working electrode was platinum disk (2 mm in diameter) for CV and RDV experiments. Saturated calomel electrode (SCE) separated by a bridge filled with supporting electrolyte and Pt wire were used as reference and auxiliary electrodes. Voltammetric measurements were performed using a potentiostat PGSTAT 128N (AUTOLAB, Metrohm Autolab B.V., Utrecht, The Netherlands) operated via NOVA 1.11 software.

3,6-Di(thiophen-2-yl)-2,5-dihydropyrrolo[3,4-*c*]pyrrol-1,4-dione (6)

Tert-amylalcohol (50 mL), sodium (1.00 g, 43.50 mmol), and catalytic amount of ferric chloride (5 mg) were added to a three-neck round-bottom flask. The mixture was heated at 145 °C under reflux until sodium was completely dissolved. Thiophene-2-carbonitrile (1.40 mL, 15.03 mmol) was added and dimethyl succinate (0,92 mL, 7,03 mmol) was added dropwise during 30 minutes. The reaction mixture was further heated at 145 °C under reflux for 2 hours, cooled to 25 °C, whereupon methanol (40 mL) and hydrochloric acid (5 mL, 35%) were added. The resulting dark red precipitate was filtered off and washed with methanol several times. Yield 1.97 g (93%); mp 300.6-301.1 °C (lit. [1] mp 300 °C). ^{1}H-NMR (400 MHz, 25 °C, d_6-DMSO): δ = 7.35 (dd, 2H, J = 4.8 Hz, J = 3.6 Hz, Th), 8.01 (dd, 2H, J = 4.8 Hz, J = 0.8 Hz, Th), 8.25 (dd, 2H, J = 3.6 Hz, J = 1.2 Hz, Th), 11.30 ppm (s, 2H, NH). ^{13}C-NMR (100 MHz, 25 °C, d_6-DMSO): δ = 108.66, 128.80, 130.83, 131.34, 132.78, 136.22, 161.69 ppm. HR-FT-MALDI-MS (DHB) *m/z*: calculated for $C_{14}H_8N_2O_2S_2^+$: 300.00217, found 300.00222 ($[M]^+$). IR (HATR): v = 2992, 2844, 2353, 1642, 1613, 1500, 1442, 1413, 1314, 1183, 1130, 855, 837, 793, 754 cm^{-1}.

***N,N'*-Dialkylated DPP derivative 7**

Diketopyrrolopyrrole derivative **6** (0.20 g, 0.67 mmol) was dissolved in *N*,*N*-dimethylformamide (100 mL) and the mixture was heated at 120 °C. Potassium carbonate (1.84 g, 13.31 mmol) was added and 2-ethylhexyl bromoacetate (3.37 g, 13.40 mmol) was added dropwise during 1 hour. The reaction mixture was further heated at 120 °C for 15 minutes and then cooled to 60 °C. DMF was evaporated and semi-solid crude product was dissolved in DCM (100 mL), washed with water (3 × 100 mL) and brine (100 mL). Combined organic extracts were dried with sodium sulfate, filtered and the solvent was evaporated. The crude product was crystallized

from methanol. Dark red solid, yield 292 mg (68%); mp 151-153 °C; R_f = 0.11 (silica gel; DCM/pentane, 4:1). 1).^{1}H-NMR (400 MHz, 25 °C, $CDCl_3$): δ = 0.79-0.84 (m, 12H, CH_3), 1.12-1.22 (m, 12H, CH_2), 1.23-1.30 (m, 4H, CH_2), 1.52-1.55 (m, 2H, CH), 4.06 (d, 4H, J = 5.6 Hz, CH_2), 4.90 (s, 4H, CH_2), 7.26 (dd, 2H, J = 5.2 Hz, J = 4.0 Hz, Th), 7.61 (dd, 2H, J = 5.2 Hz, J = 1.2 Hz, Th), 8.76 ppm (dd, 2H, J = 4.0 Hz, J = 1.2 Hz, Th). ^{13}C-NMR (100 MHz, 25 °C, $CDCl_3$): δ = 11.09, 14.20, 23.12, 23.82, 28.99, 30.45, 38.81, 43.86, 68.39, 10.80, 129.11,129.73, 131.02, 135.36, 140.07, 161.15, 168.44 ppm. HR-FT-MALDI-MS (DHB) m/z: calculated for $C_{34}H_{44}N_2O_6S_2^+$: 640.26353, found 640.26409 ($[M]^+$). IR (HATR): v = 2957, 2925, 2860, 2356, 1743, 1651, 1568, 1498, 1460, 1419, 1407, 1338, 1272, 1227, 1192, 1123, 1088, 1028, 974, 857, 740, 715, 689 cm^{-1}.

Dibromo DPP derivative 8

Diketopyrrolopyrrole derivative **7** (1.12 g, 1.75 mmol) was dissolved in DCM (100 mL). *N*-Bromosuccinimide (622 mg, 3.50 mmol) and acetic acid (0.3 mL) were added. The reaction was stirred at 25 °C for 2 hours and quenched by water (100 mL). The product was extracted with DCM (3 × 100 mL), the combined organic extracts were dried with sodium sulfate, filtered and the solvent was evaporated. The crude product was purified by column chromatography (silica gel, DCM). Dark red solid, yield 1.30 g (93%); mp 152-153 °C; R_F = 0.42 (silica gel, DCM/pentane, 4:1). ^{1}H-NMR (400 MHz, 25 °C, $CDCl_3$): δ = 0.82-0.86 (m, 12H, CH_3), 1.20-1.24 (m, 12H, CH_2), 1.26-1.34 (m, 4H, CH_2), 4.09 (d, 2H, J = 5.6 Hz, CH_2), 4.09 (d, 2H, J = 5.6 Hz, CH_2), 4.81 (s, 4H, CH_2), 7.21 (d, 2H, J = 4.0 Hz, Th), 8.48 ppm (d, 2H, J = 4.4 Hz, Th). ^{13}C-NMR (100 MHz, 25 °C,$CDCl_3$): δ = 11.14; 14.27; 23.16; 23.86; 29.05; 30.49; 38.87; 43.80; 68.59; 107.95; 119.70; 131.10; 132.16; 135.42; 139.03; 160.86; 168.21 ppm. HR-FT-MALDI-MS (DHB) m/z: calculated for $C_{34}H_{42}Br_2N_2O_6S_2^+$: 798.08251,

found 798.08426 ($[M]^+$). IR (HATR): v = 3094, 2952, 2922, 2856, 1746, 1662, 1561, 1504, 1458, 1421, 1408, 1395, 1318, 1261, 1229, 1201, 1133, 1036, 1005, 974, 887, 839, 802, 736, 697 cm^{-1}.

General method for Suzuki–Miyaura cross-coupling reaction

Diketopyrrolopyrrole derivative **8** (200 mg, 0.25 mmol), the appropriate boronic acid (0.53 mmol), tetrahydrofuran (80 mL) and water (20 mL) were added to a flame-dried Schlenk flask. The solution was bubbled with argon for 5 minutes. Sodium carbonate (0.25 mmol) and $PdCl_2(PPh_3)_2$ (0.013 mmol) were added and the reaction was heated at 65 °C for 24 hours. The product was extracted with DCM (100 mL), the organic extract was washed with water (3 × 100 mL), dried with sodium sulfate, filtered and the solvents were evaporated. The crude product was purified by column chromatography (silica gel, DMC/acetone, 50:1) and subsequent crystallization from a mixture of DCM and hexane.

Chromophore 1a

The title compound was synthesized from **8** and 4-(*N*,*N*-dimethylamino)phenylboronic acid (87 mg, 0.53 mmol) following the general procedure. Dark green solid. Yield 82 mg (37%); mp 262-263 °C; R_F = 0.28 (silica gel, DCM/acetone, 50:1). ^{1}H-NMR (400 MHz, 25 °C, $CDCl_3$): δ = 0.78-0.82 (m, 12H, CH_3), 1.19-1.22 (m, 12H, CH_2), 1.26-1.32 (m, 4H, CH_2), 1.51-1.54 (m, 2H, CH), 3.01 (s, 12H, *N*-CH_3), 4.08 (d, 2H, J = 5.6 Hz, CH_2), 4.08 (d, 2H, J = 5.6 Hz, CH_2), 4.97 (s, 4H, CH_2), 6.69 (d, 4H, J = 8.8 Hz, ArH), 7.29 (d, 2H,J = 4.4 Hz, Th), 7.52 (d, 4H, J = 8.8 Hz, ArH), 8.82 ppm (d, 2H, J = 4.4 Hz, Th).^{13}C-NMR (125 MHz, 25 °C,$CDCl_3$): δ = 11.12; 14.26; 23.14; 23.82; 29.03; 30.46; 38.80; 40.49; 40.70; 43.99; 68.28; 107.21; 112.36; 122.71; 126.53; 127.47; 137.17; 139.04; 151.00; 151.75; 161.21; 168.74 ppm. HR-FT-MALDI-MS (DHB) *m/z*: calculated for $C_{50}H_{62}N_4O_6S_2^+$: 878.41053, found 878.41248 ($[M]^+$). IR

(HATR): v = 3282, 2361, 1746, 1659, 1604, 1558, 1500, 1424, 1393, 1364, 1334, 1192, 1118, 1096, 1033, 806 cm^{-1}.

Chromophore 2a

The title compound was synthesized from **8** and 4-methoxyphenylboronic acid (80 mg, 0.53 mmol) following the general procedure. Dark green solid. Yield 118 mg (55%); mp 245-247 °C; R_F = 0.34 (silica gel, DCM/acetone, 50:1). ^{1}H-NMR (500 MHz, 25 °C, $CDCl_3$): δ = 0.78-0.81 (m, 12H, CH_3), 1.18-1.22 (m, 12H, CH_2), 1.25-1.31 (m, 4H, CH_2), 1.52-1.54 (m, 2H, CH), 3.85 (s, 6H, *O*-CH_3), 4.08 (d, 2H, *J* = 4.8 Hz, CH_2), 4.09 (d, 2H, *J* = 4.4 Hz, CH_2), 4.96 (s, 4H, CH_2), 6.92 (d, 4H, *J* = 8.5 Hz, ArH), 7.35 (d, 2H, *J* = 4.0 Hz, Th), 7.58 (d, 4H, *J* = 9.0 Hz, ArH), 8.81 ppm (d, 2H, *J* = 4.0 Hz, Th). ^{13}C-NMR (100 MHz, 25 °C,$CDCl_3$): δ = 11.10; 14.15; 23.06; 23.81; 28.98; 30.42; 38.81; 43.93; 55.59; 68.32; 107.66; 114.71; 124.02; 125.86; 127.74; 136.86; 139.30; 150.43; 160.57; 161.13; 168.52 ppm. HR-FT-MALDI-MS (DHB) *m/z*: calculated for $C_{48}H_{56}N_2O_8S_2^+$: 852.34726, found 852.34896 ($[M]^+$). IR (HATR): v = 2961, 2927, 2341, 1746, 1661, 1605, 1562, 1496, 1434, 1411, 1296, 1255, 1201, 1181, 1120, 1094, 1035, 978, 833, 802, 742 cm^{-1}.

Chromophore 3a

The title compound was synthesized from **8** and 2-thienylboronic acid (67 mg, 0.53 mmol) following the general procedure. Dark green solid. Yield 93 mg (46%); mp 208-210 °C; R_F = 0.36 (silica gel, DCM/acetone, 50:1). ^{1}H-NMR (400 MHz, 25 °C, $CDCl_3$): δ = 0.80-0.83 (m, 12H, CH_3), 1.19-1.24 (m, 12H, CH_2), 1.26-1.33 (m, 4H, CH_2), 4.09 (d, 2H, *J* = 5.6 Hz, CH_2), 4.09 (d, 2H, *J* = 5.6 Hz, CH_2), 4.93 (s, 4H, CH_2), 7.06 (dd, 2H, *J* = 5.2 Hz, *J* = 3.6 Hz, Th), 7.29-7.33 (m, 6H, Th), 8.76 ppm (d, 2H, *J* = 5.2 Hz, Th). ^{13}C-NMR (100 MHz, 25 °C,$CDCl_3$): δ = 11.07; 14.16; 23.07; 23.81; 28.99; 30.43; 38.81; 43.87; 68.39; 108.11; 125.38; 125.65; 126.74; 127.97; 128.45;

136.01; 136.60; 139.11; 143.32; 161.04; 168.40 ppm. HR-FT-MALDI-MS (DHB) *m/z*: calcualted for $C_{42}H_{48}N_2O_6S_4^+$: 804.23897, found 804.24030 ($[M]^+$). IR (HATR): v = 2957, 2922, 2852, 1737, 1665, 1556, 1537, 1439, 1424, 1358, 1316, 1222, 1197, 1118, 1029, 974, 844, 806, 758, 738, 705 cm^{-1}.

Chromophore 4a

Diketopyrrolopyrrole derivative **8** (586 mg, 0.73 mmol) was dissolved in dry tetrahydrofuran (100 mL) in a flame-dried Schlenk flask. Tributyl-(5-methoxythiophen-2-yl)stannane (600 mg, 1.49 mmol) was added and the solution was bubbled with argon for 5 minutes. $PdCl_2(PPh_3)_2$ (26 mg, 0.04 mmol) was added and the reaction mixture was heated to 65 °C for 16 hours and quenched with water (100 mL). The product was exctracted with DCM (100 mL), the organic extract was dried with soudium sulfate, filtered and the solvent was evaporated. The crude product was purified by column chromatography (silica gel, DMC/acetone, 50:1) and subsequent crystallization from a mixture of DCM and hexane. Dark green solid. Yield 307 mg (48%); mp 198-200 °C; R_F = 0.35 (silica gel, DCM/acetone, 50:1). ^{1}H-NMR (400 MHz, 25 °C, $CDCl_3$): δ = 0.81 (t, 12H, *J*= 7.6 Hz, CH_3), 1.20-1.25 (m, 12H, CH_2), 1.27-1.32 (m, 4H, CH_2), 1.51-1.54 (m, 2H, CH), 3,92 (s, 6H, *O*-CH_3), 4.08 (dd, 4H, *J*= 5.6 Hz, *J*= 1.6 Hz, *O*-CH_2), 4.91 (s, 4H, *N*-CH_2), 6.15 (d, 2H, *J*= 4.0 Hz, Th), 6.95 (d, 2H, *J*= 4.0 Hz, Th), 7.08 (d, 2H, *J*= 4.4 Hz, Th), 8.72 ppm (d, 2H, *J*= 4.0 Hz, Th). ^{13}C-NMR (100 MHz, 25 °C, $CDCl_3$): δ = 11.12; 14.23; 23.13; 23.86; 29.04; 30.47; 38.84; 43.89; 60.57; 68.35; 105.29; 107.71; 122.49; 123.63; 123.96; 126.77; 136.66; 138.71; 144.21; 160.99; 167.76; 168.52 ppm. HR-FTMALDI-MS (DHB) *m/z*: calculated for $C_{44}H_{52}N_2O_8S_4^+$: 864.26010, found 864.26195 ($[M]^+$). IR (HATR): v = 3072, 2924, 2859, 1741, 1656, 1550, 1499, 1466, 1417, 1252, 1181, 1116, 1063, 1023, 991, 815, 767, 735, 687, 634, 572, 479 cm^{-1}.

General method for Sonogashira cross-coupling reaction

Diketopyrrolopyrrole derivative **8** (300 mg, 0.38 mmol), an appropriate terminal acetylene (0.79 mmol), dry tetrahydrofuran (150 mL), and triethylamine (2 mL) were added to a flame-dried Schlenk flask. The solution was bubbled with argon for 5 minutes whereupon copper(I) iodide (0.02 mmol) and $PdCl_2(PPh_3)_2$ (0.02 mmol) were added. The reaction mixture was heated to 65 °C for 24 hours and subsequently extracted with DCM (100 mL). The organic extract was washed with water (3 × 100 mL), dried with sodium sulfate, filtered, and the solvents were evaporated. The crude product was purified by column chromatography (silica gel, DMC) and subsequent crystallization from a mixture of DCM and hexane.

Chromophore 1b

The title compound was synthesized from **8** and 4-ethynyl-*N*,*N*-dimethylaniline (115 mg, 0.79 mmol) following the general procedure. Dark brown solid. Yield 220 mg (63%); mp 223-225 °C; R_F = 0.42 (silica gel, DCM). ^{1}H-NMR (400 MHz, 25 °C, $CDCl_3$): δ = 0.81-0.85 (m, 12H, CH_3), 1.21 (br s, 12H, CH_2), 1.26-1.31 (m, 4H, CH_2), 3.00 (s, 12H, *N*-CH_3), 4.09 (d, 2H, J = 5.6 Hz, CH_2), 4.09 (d, 2H, J = 5.6 Hz, CH_2), 4.91 (s, 4H, CH_2), 6.65 (d, 4H, J = 8.8 Hz, ArH), 7.28 (d, 2H, J = 4.0 Hz, Th), 7.38 (d, 4H, J = 8.8 Hz, ArH), 8.80 ppm (d, 2H, J = 4.0 Hz, Th). ^{13}C-NMR (100 MHz, 25 °C,$CDCl_3$): δ = 11.05; 14.14; 23.07; 23.83; 28.98; 30.44; 38.81; 40.23; 43.91; 68.41; 80.88; 100.62; 108.27; 108.72; 111.89; 129.21; 130.61; 132.18; 135.77; 138.90; 150.74; 160.98; 168.30 ppm. HR-FT-MALDI-MS (DHB) *m/z*: calculated for $C_{54}H_{62}N_4O_6S_2^+$: 926.41053, found 926.41126 ($[M]^+$). IR (HATR): ν = 2957, 2928, 2184, 1749, 1662, 1602, 1554, 1530, 1432, 1414, 1366, 1300, 1240, 1193, 1126, 1020, 944, 915, 883, 815, 757, 738 cm^{-1}.

Chromophore 2b

The title compound was synthesized from **8** and 4-ethynylanisole (104 mg, 0.79 mmol) following the general procedure. Dark brown solid. Yield 186 mg (55%); mp 177-178 °C; R_F = 0.28 (silica gel, acetone/pentane, 1:5). ^{1}H-NMR (400 MHz, 25 °C, $CDCl_3$): δ = 0.80-0.85 (m, 12H, CH_3), 1.21 (br s, 12H, CH_2), 1.26-1.33 (m, 4H, CH_2), 3.83 (s, 6H, *O*-CH_3), 4.09 (d, 2H, *J* = 5.6 Hz, CH_2), 4.10 (d, 2H, *J* = 5.6 Hz, CH_2), 4.91 (s, 4H, CH_2), 6.89 (d, 4H, *J* = 8.8 Hz, ArH), 7.32 (d, 2H, *J* = 4.0 Hz, Th), 7.45 (d, 4H, *J* = 8.8 Hz, ArH), 8.78 ppm (d, 2H, *J* = 4.0 Hz, Th). ^{13}C-NMR (100 MHz, 25 °C,$CDCl_3$): δ = 11.02; 14.13; 23.04; 23.75; 28.93; 30.37; 38.73; 43.84; 55.47; 68.40; 81.10; 98.68; 108.42; 114.24; 114.32; 129.72; 129.76; 132.85; 133.26; 135.65; 138.98; 160.45; 160.89; 168.23 ppm. HR-FT-MALDI-MS (DHB) *m/z*: calculated for $C_{52}H_{56}N_2O_8S_2^+$: 900.34726, found 900.34884 ($[M]^+$).IR (HATR): v = 2953, 2926, 2198, 1753, 1668, 1603, 1563, 1520, 1458, 1432, 1415, 1288, 1251, 1199, 1174, 1128, 1114, 1027, 976, 879, 830, 818, 744, 721, 691 cm^{-1}.

Chromophore 3b

The title compound was synthesized from **8** and 2-ethynylthiophene (85 mg, 0.79 mmol) following the general procedure. Dark green solid. Yield 144 mg (45%); mp 159-164 °C; R_F = 0.45 (silica gel, DCM). ^{1}H-NMR (400 MHz, 25 °C, $CDCl_3$): δ = 0.81-0.86 (m, 12H, CH_3), 1.22 (br s, 12H, CH_2), 1.27-1.34 (m, 4H, CH_2), 4.10 (d, 2H, *J* = 5.6 Hz, CH_2), 4.10 (d, 2H, *J* = 5.6 Hz, CH_2), 4.90 (s, 4H, CH_2), 7.04 (do, 2H, *J* = 5.2 Hz, *J* = 3.6 Hz, Th), 7.32 (do, 2H, *J* = 3.6 Hz, *J* = 0.8 Hz, Th), 7.37 (do, 4H, *J* = 5.2 Hz, *J* = 0.8 Hz, Th), 8.79 ppm (d, 2H, *J* = 3.6 Hz, Th). ^{13}C-NMR (100 MHz, 25 °C,$CDCl_3$): δ = 11.15; 14.25; 23.17; 23.88; 29.05; 30.49; 38.86; 43.95; 68.58; 85.90; 91.75; 108.79; 122.22; 127.66; 128.85; 129.03; 130.51; 13.25; 133.44; 135.75; 139.12; 160.99; 168.29 ppm. HR-FT-MALDI-MS (DHB) *m/z*: calculated for

$C_{46}H_{48}N_2O_6S_4^+$: 852.23897, found 852.23994 ($[M]^+$). IR (HATR): *v* = 3085, 2953, 2919, 2182, 1745, 1660, 1557, 1440, 1409, 1359, 1324, 1236, 1199, 1120, 1106, 1059, 1013, 976, 851, 814, 705, 694 cm^{-1}.

Chromophore 4b

The title compound was synthesized from **8** and 2-ethynyl-5-methoxythiophene (109 mg, 0.79 mmol) following the general procedure. Dark brown solid. Yield 110 mg (32%); mp 144-146 °C; R_F = 0.46 (silica gel, DCM). ^{1}H-NMR (400 MHz, 25 °C, $CDCl_3$): δ = 0.81-0-85 (m, 12H, CH_3), 1.21-1.23 (m, 12H, CH_2), 1.26-1.33 (m, 4H, CH_2), 1.51 (br s, 2H, CH), 3.92 (s, 6H, *O*-CH_3), 4.09 (d, 2H, *J* = 6.0 Hz, CH_2), 4.09 (d, 2H, *J* = 5.6 Hz, CH_2), 4.89 (s, 4H, CH_2), 6.13 (d, 2H, *J* = 4.0 Hz, Th), 7.00 (d, 2H, *J* = 4.0 Hz, Th), 7.30 (d, 2H, *J* = 4.4 Hz, Th), 8.78 ppm (d, 2H, *J* = 4.4 Hz, Th). ^{13}C-NMR (100 MHz, 25 °C,$CDCl_3$): δ = 11.08; 14.18; 23.10; 23.82; 28.98; 30.43; 38.79; 43.88; 60.48; 68.48; 84.11; 92.84; 104.76; 108.27; 108.56; 129.28; 129.95; 132.41; 132.85; 135.72; 138.94; 160.93; 168.25; 168.72 ppm. HR-FT-MALDI-MS (DHB) *m/z*: calculated for $C_{48}H_{52}N_2O_8S_4^+$: 912.26010, found 912.26112 ($[M]^+$). IR (HATR): *v* = 2953, 2921, 2852, 2175, 1750, 1667, 1557, 1542, 1507, 1477, 1407, 1326, 1290, 1198, 1126, 1107, 1078, 1007, 989, 974, 879, 822, 780, 740, 719, 689 cm^{-1}.

Chromophore 5b

The title compound was synthesized from **8** and ethynylferrocene (166 mg, 0.79 mmol) following the general procedure without crystallization. Dark brown solid. Yield 290 mg (73%); mp 189-191 °C; R_F = 0.76 (silica gel, DCM). ^{1}H-NMR (400 MHz, 25 °C, $CDCl_3$): δ = 0.83-0.86 (m, 12H, CH_3), 1.22 (br s, 12H, CH_2), 1.29-1.32 (m, 4H, CH_2), 4.10 (d, 4H, *J* = 2.8 Hz, CH_2), 4.26 (s, 10H, CpH), 4.32 (s, 4H, CpH), 4.53 (s, 4H, CpH), 4.91 (s, 4H, CH_2), 7.29 (d, 2H, *J* = 4.0 Hz, Th), 8.74 ppm (d, 2H, *J* = 4.0

Hz, Th). ^{13}C-NMR (100 MHz, 25 °C, $CDCl_3$): δ = 11.09; 14.21; 23.09; 23.81; 28.97; 30.42; 38.76; 43.89; 64.23; 68.41; 70.07; 70.62; 71.94; 78.77; 99.04; 108.39; 129.34; 130.21; 132.56; 135.63; 138.88; 160.95; 168.34 ppm. HR-FT-MALDI-MS (DHB) *m/z*: calculated for $C_{58}H_{60}N_2O_6S_2Fe_2^+$: 1056.25861, found 1056.25944 ($[M]^+$). IR (HATR): ν = 2357, 2187, 1749, 1669, 1569, 1401, 1237, 1191, 1116, 1017, 814 cm^{-1}.

THG measurement's method

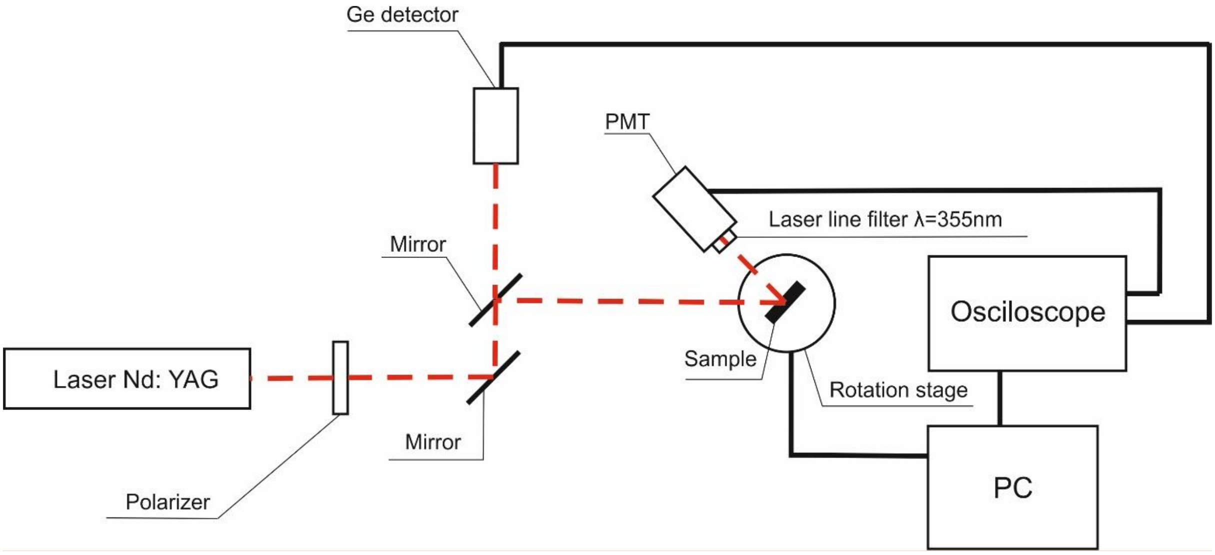

Figure S1: Principal set-up for the measurements of the THG in the reflected light geometry.

The 10 ns Nd:YAG laser was used as a source of the fundamental laser beam at wavelength 1064 nm. The pulse duration was about 10 ns, the pulse frequency repetition was equal to about 11 Hz. The output laser energy was tuned by rotating polarizer. The set-up allows to achieve the maximal energy density of about 199.05 J/m^2. The beam dimer was varied within the 1.5 to 3.5 mm and its beam sequence was of a Gaussian-like form. The stability of the pulse was averaged over 200 pulses and did not exceed 0.4%. Its energy was controlled by Ge detector with 0.8 ns relaxation time. The fundamental beam has illuminated the sample's surface using a set of mirrors. The chromophores **1**–**5** were placed between two glass plates. The reflected light from the chromophore was analyzed using an interferometric filter at 355 nm and photomultiplier. The samples were put on a rotating table in order to find the maximal THG signal, which was analyzed with 2 GHz Tectronix oscilloscope. The measurements were operated by a PC. The control of the sample surfaces has shown that the local heating did not exceed 2 to 3 K. Particular attention was devoted

to elimination of the parasitic light scattering and this background did not exceed 0.6%.

The THG value was evaluated by the effective surfaces under the curves shown in the Figure 6 after the statistical averagment over 200 different surface points. The further increase of fundamental laser energy was limited by occurrence of specimen's photo-destruction. Higher THG responses was observed for **1a** and **4b**, however due to their high linear absorption at 355 nm, the renormalization for the absorption was done and the resulted THG magnitudes were different. The BiB_3O_6 crystals were used as reference samples [2]. The absolute values are given in the Table 2, the reproducibility of the data was better than 2.3%.

References

1. Stas S.; Sergeyev S; Geerts Y. *Tetrahedron* **2010**, *66*, 1837-1845.
2. Ghotbi, M.; Sun, Z.; Majchrowski, A.; Michalski, E.; Kityk. I. V. *Appl. Phys. Lett.* **2006**, *89*, (2006), 173124.
