# Supplementary material for: Structure–property relationships and third-order nonlinearities in diketopyrrolopyrrole based D–π–A–π–D molecules
Source: Beilstein J Org Chem. 2017 Nov 8;13:2374–84. doi: 10.3762/bjoc.13.235 (PMC5687008; doi:10.3762/bjoc.13.235)

Supporting Information File 2

for

Structure–property relationships and third-order nonlinearities in diketopyrrolopyrrole based D–π–A–π–D molecules

Jan Podlesný[1], Lenka Dokládalová[2], Oldřich Pytela[1], Adam Urbanec[1], Milan Klikar[1], Numan Almonasy[1], Tomáš Mikysek[3], Jaroslav Jedryka[4], Iwan V. Kityk[4] and Filip Bureš*[1]

Address: [1]Institute of Organic Chemistry and Technology, Faculty of Chemical Technology, University of Pardubice, Studentská 573, Pardubice, 53210, Czech Republic, [2]Centre of Organic Chemistry Ltd., Rybitví 296, Rybitví, 53354, Czech Republic, [3]Department of Analytical Chemistry, Faculty of Chemical Technology, University of Pardubice, Studentská 573, Pardubice, 53210, Czech Republic, and [4]Institute of Optoelectronics and Measuring Systems, Faculty of Electrical Engineering, Czestochowa University of Technology, Armii Krajowej 17, Czestochowa, 42-200, Poland

Email: Filip Bureš - filip.bures@upce.cz

* Corresponding author

^{1}H and ^{13}C NMR spectra, HR-MALDI-MS spectra, CV curves, UV–vis absorption/emission spectra, and HOMO/LUMO localizations

Table of contents

1. ^{1}H and ^{13}C NMR and HR-MALDI-MS spectra of chromophore **1a** S3
2. ^{1}H and ^{13}C NMR and HR-MALDI-MS spectra of chromophore **2a** S5
3. ^{1}H and ^{13}C NMR and HR-MALDI-MS spectra of chromophore **3a** S7
4. ^{1}H and ^{13}C NMR and HR-MALDI-MS spectra of chromophore **4a** S9
5. ^{1}H and ^{13}C NMR and HR-MALDI-MS spectra of chromophore **1b** S11
6. ^{1}H and ^{13}C NMR and HR-MALDI-MS spectra of chromophore **2b** S13
7. ^{1}H and ^{13}C NMR and HR-MALDI-MS spectra of chromophore **3b** S15
8. ^{1}H and ^{13}C NMR and HR-MALDI-MS spectra of chromophore **4b** S17
9. ^{1}H and ^{13}C NMR and HR-MALDI-MS spectra of chromophore **5b** S19
10. Electrochemistry S21
11. Electronic absorption and emission spectra S24
12. HOMO and LUMO visualization S26

1. ^{1}H and ^{13}C NMR and HR-MALDI-MS spectra of chromophore 1a

^{1}H NMR spectrum (400 MHz, $CDCl_3$, 25 °C) of **1a**

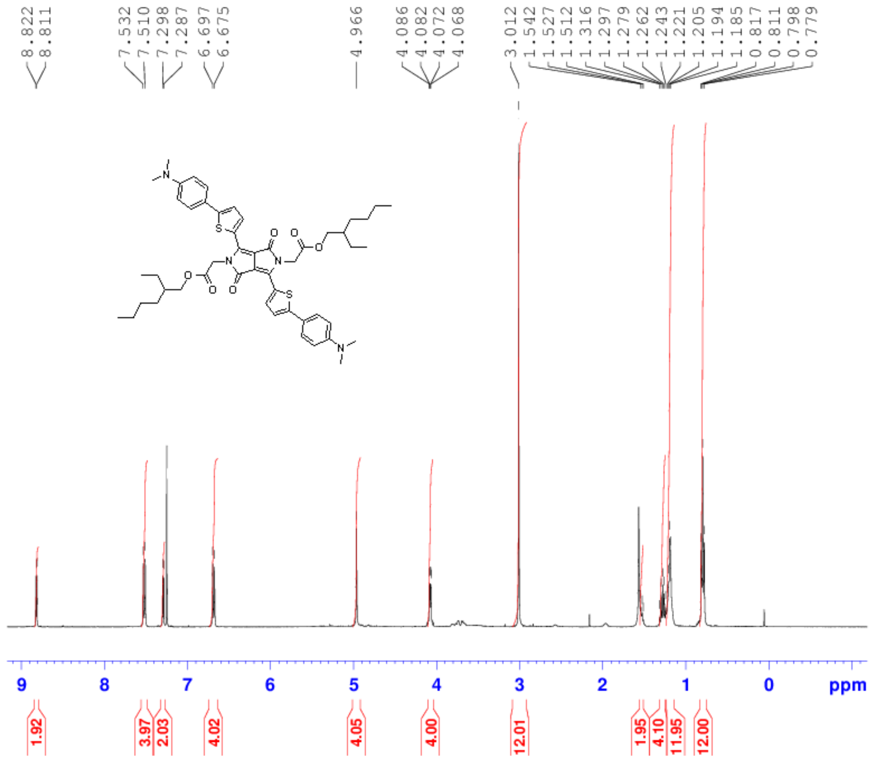

^{13}C NMR spectrum (125 MHz, $CDCl_3$, 25 °C) of **1a**

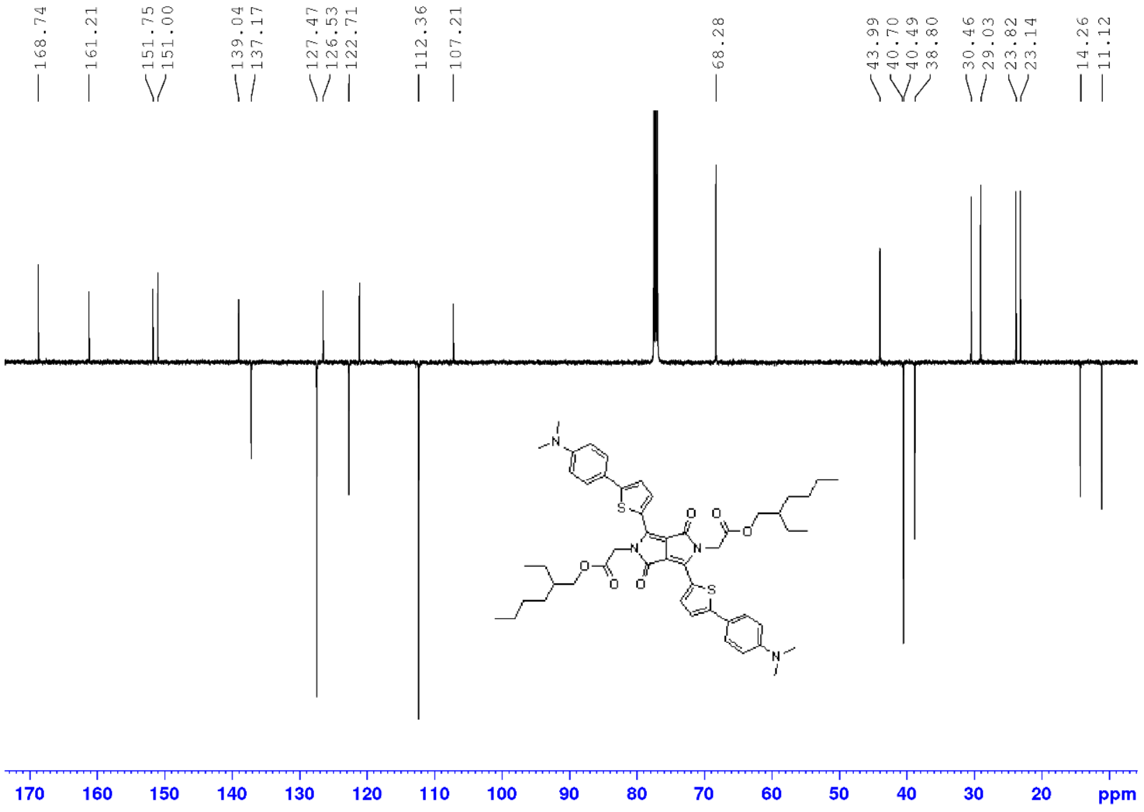

Experimental (up) and calculated (down) MALDI spectra of **1a**

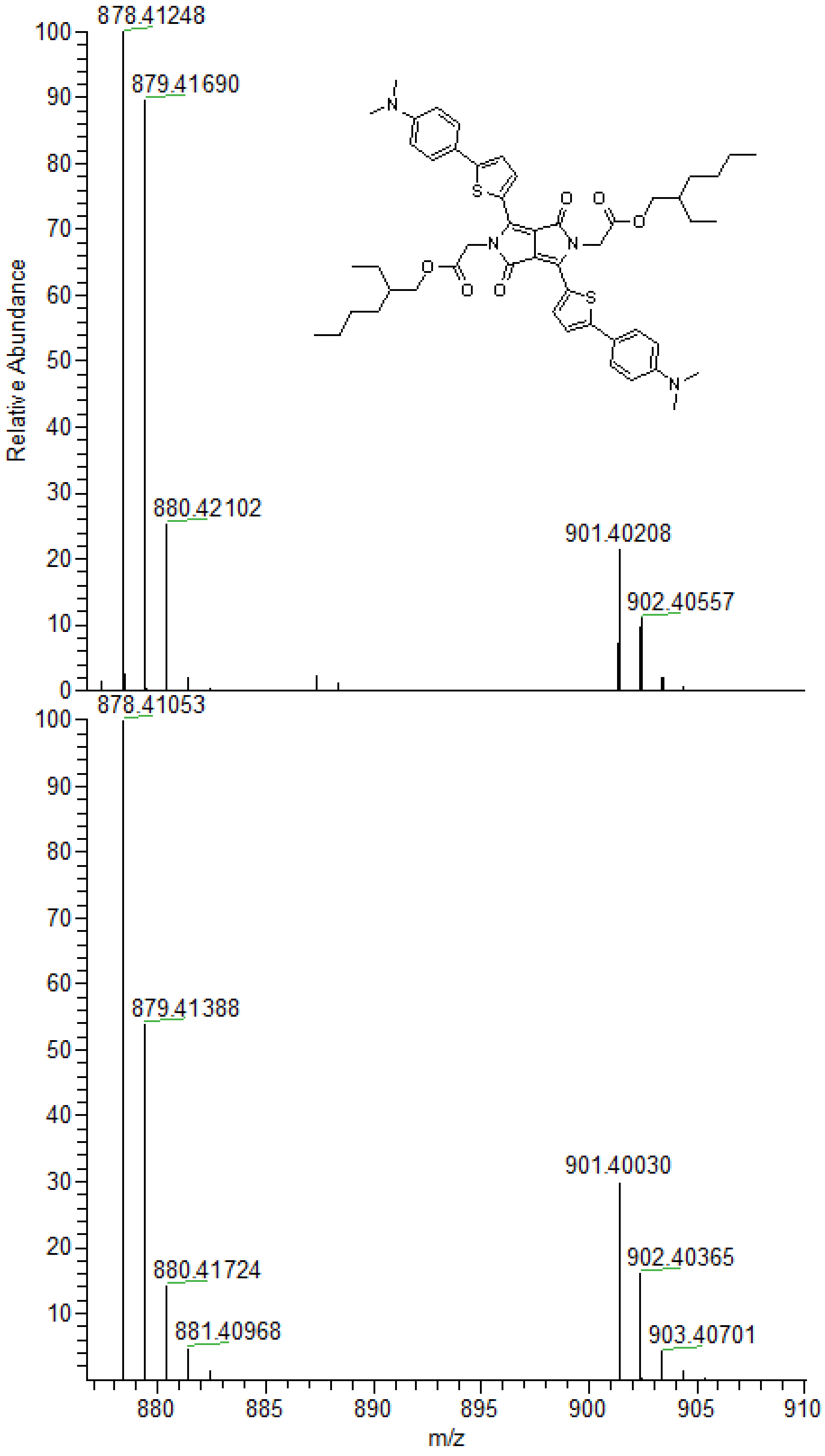

2. ^{1}H and ^{13}C NMR and HR-MALDI-MS spectra of chromophore 2a

^{1}H NMR spectrum (500 MHz, $CDCl_3$, 25 °C) of **2a**

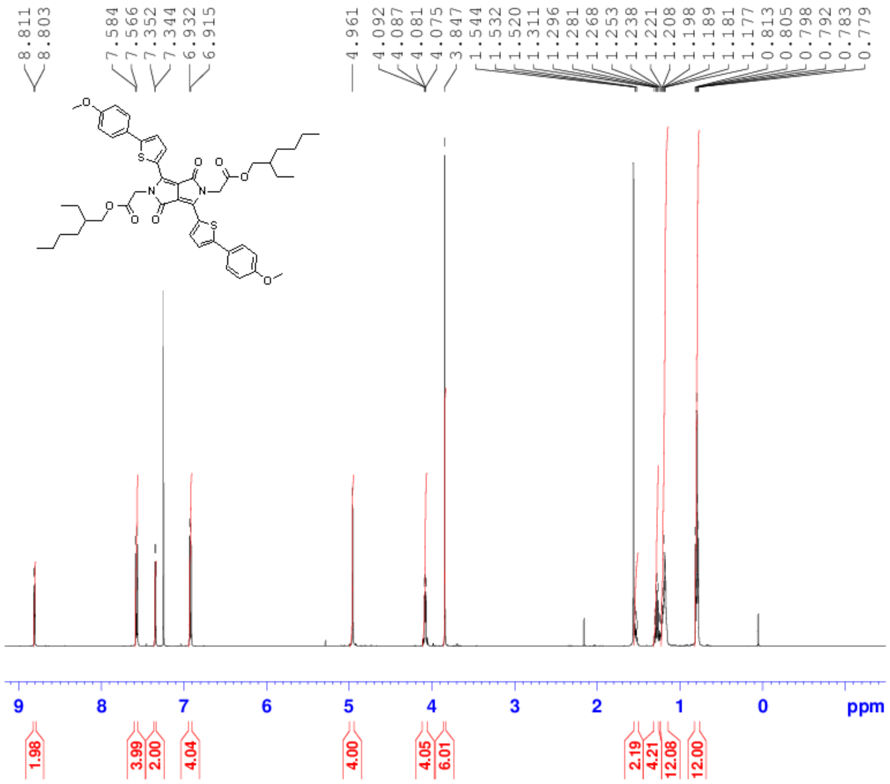

^{13}C NMR spectrum (100 MHz, $CDCl_3$, 25 °C) of **2a**

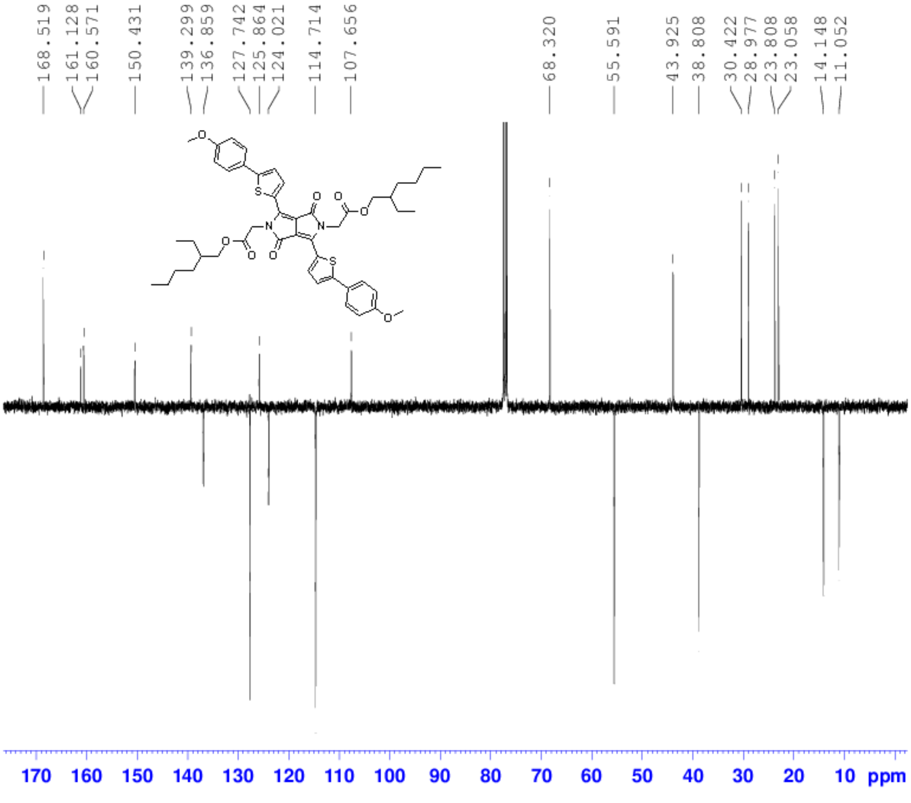

Experimental (up) and calculated (down) MALDI spectra of **2a**

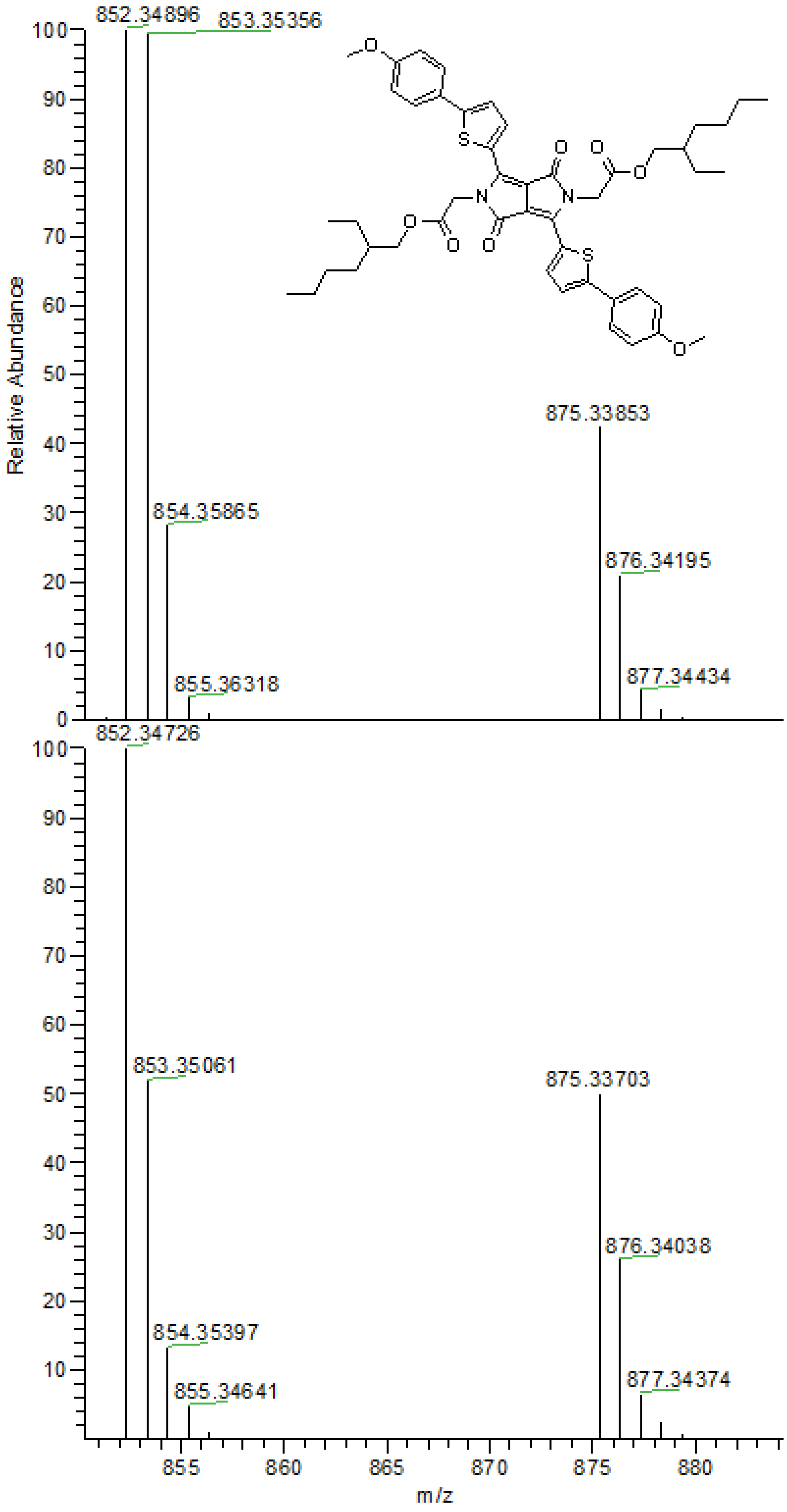

3. ^{1}H and ^{13}C NMR and HR-MALDI-MS spectra of chromophore 3a

^{1}H NMR spectrum (400 MHz, $CDCl_3$, 25 °C) of **3a**

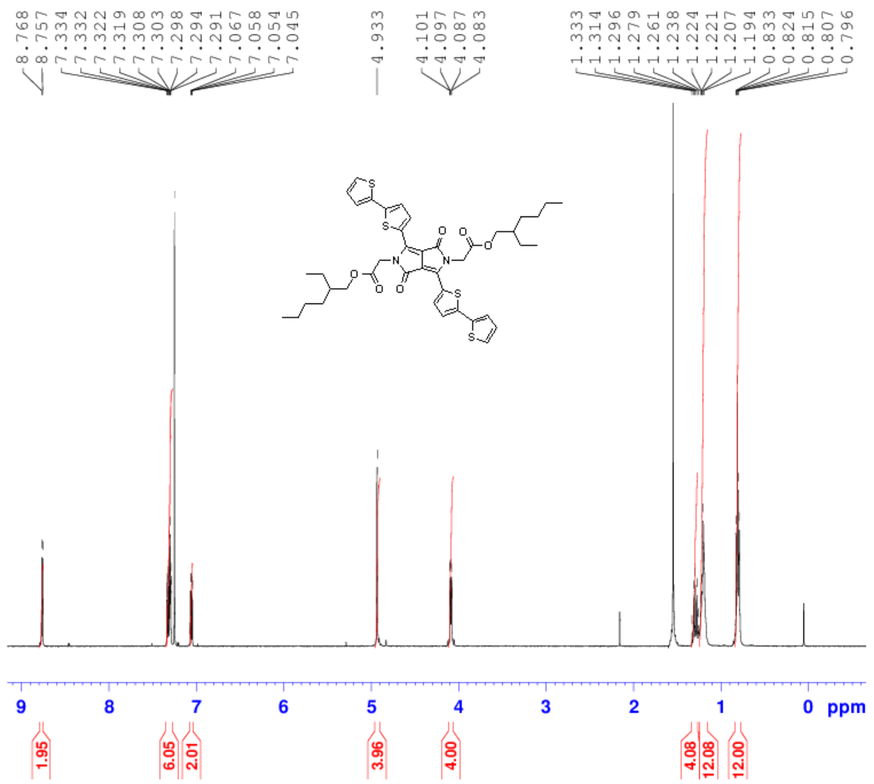

^{13}C NMR spectrum (100 MHz, $CDCl_3$, 25 °C) of **3a**

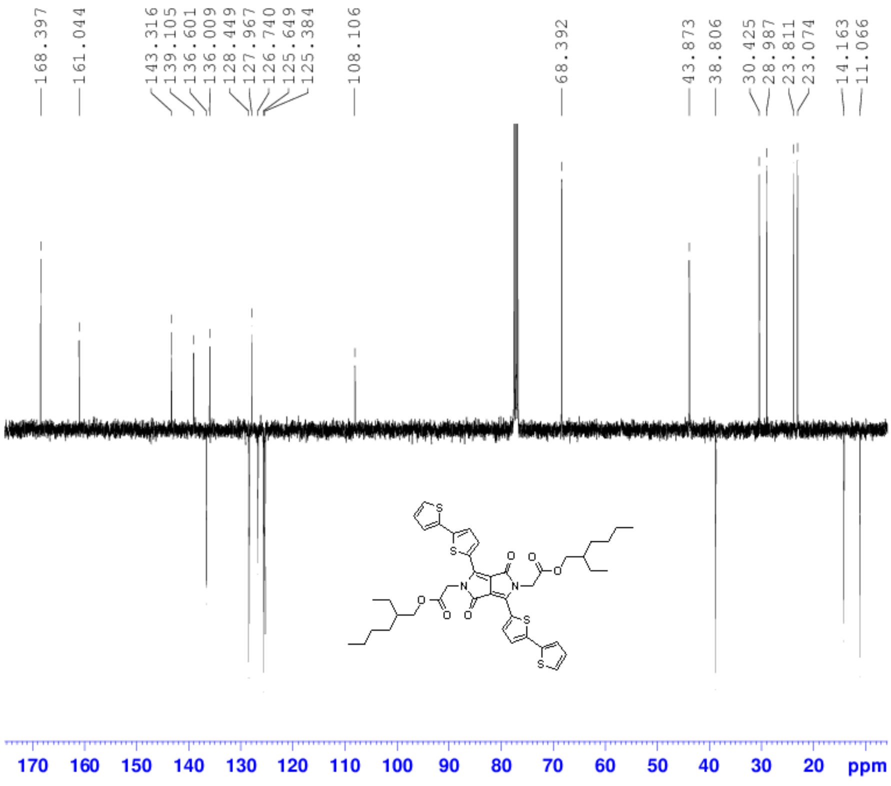

Experimental (up) and calculated (down) MALDI spectra of **3a**

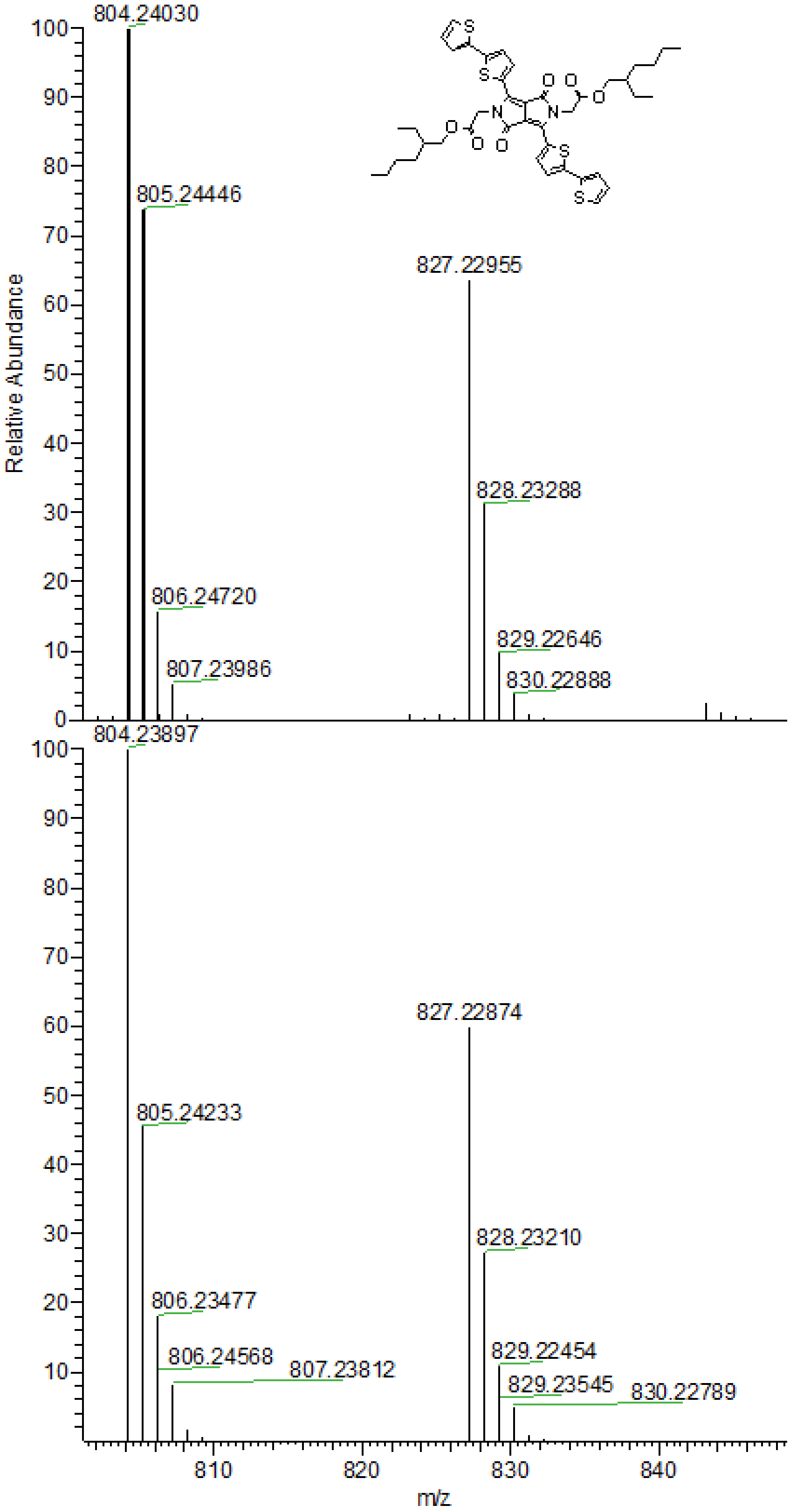

4. ^{1}H and ^{13}C NMR and HR-MALDI-MS spectra of chromophore 4a

^{1}H NMR spectrum (400 MHz, $CDCl_3$, 25 °C) of **4a**

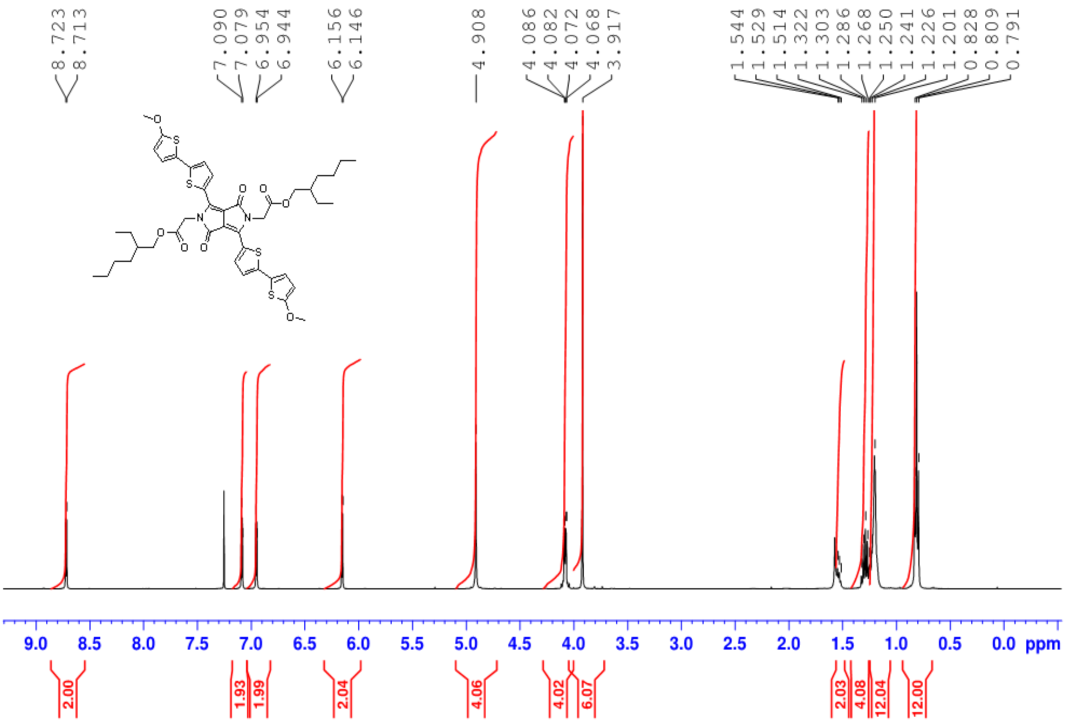

^{13}C NMR spectrum (100 MHz, $CDCl_3$, 25 °C) of **4a**

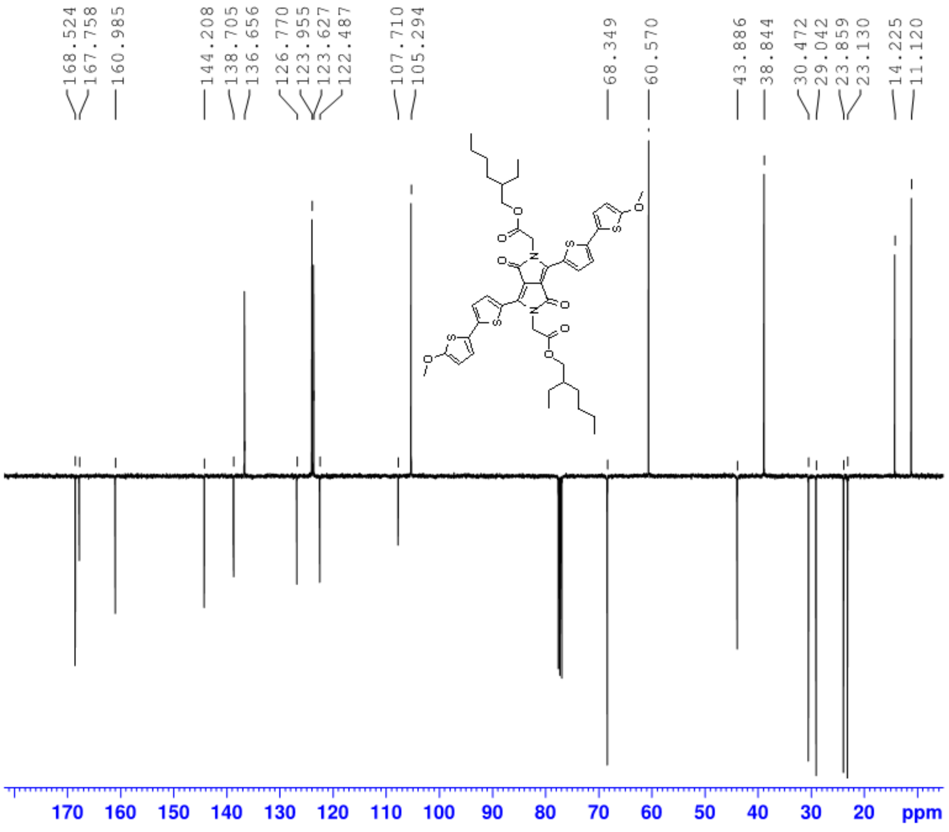

Experimental (up) and calculated (down) MALDI spectra of **4a**

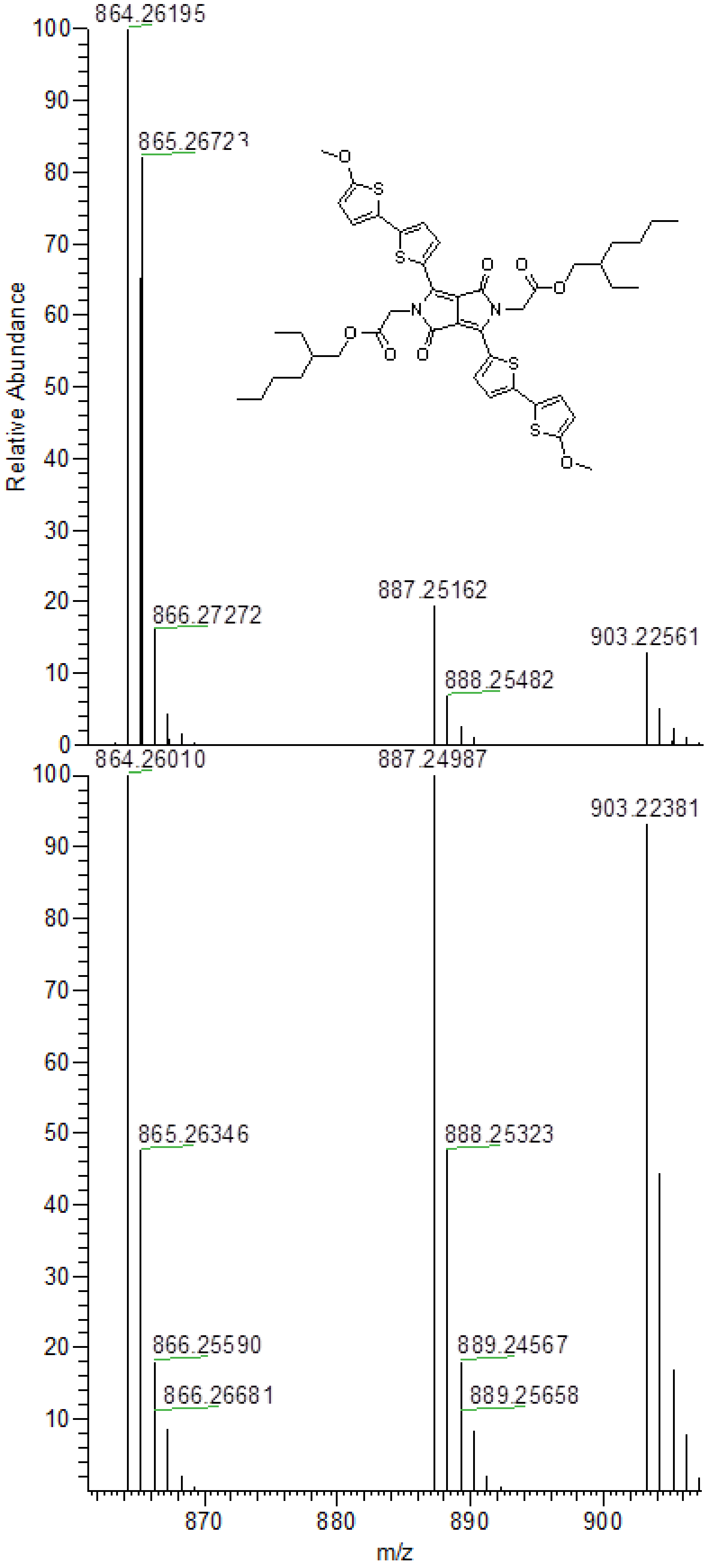

5. ^{1}H and ^{13}C NMR and HR-MALDI-MS spectra of chromophore 1b

^{1}H NMR spectrum (400 MHz, $CDCl_3$, 25 °C) of **1b**

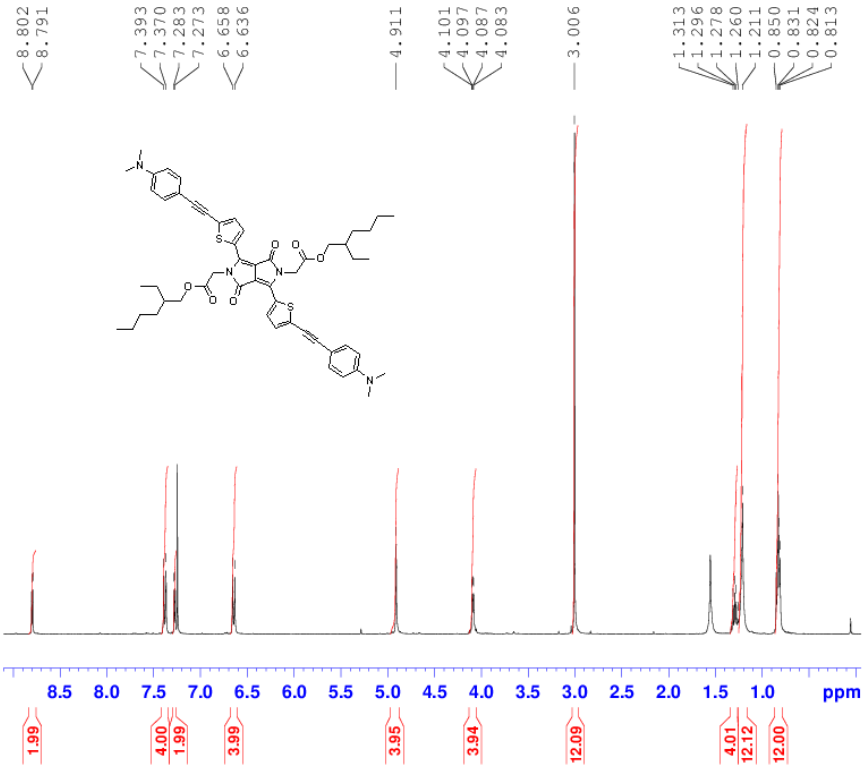

^{13}C NMR spectrum (100 MHz, $CDCl_3$, 25 °C) of **1b**

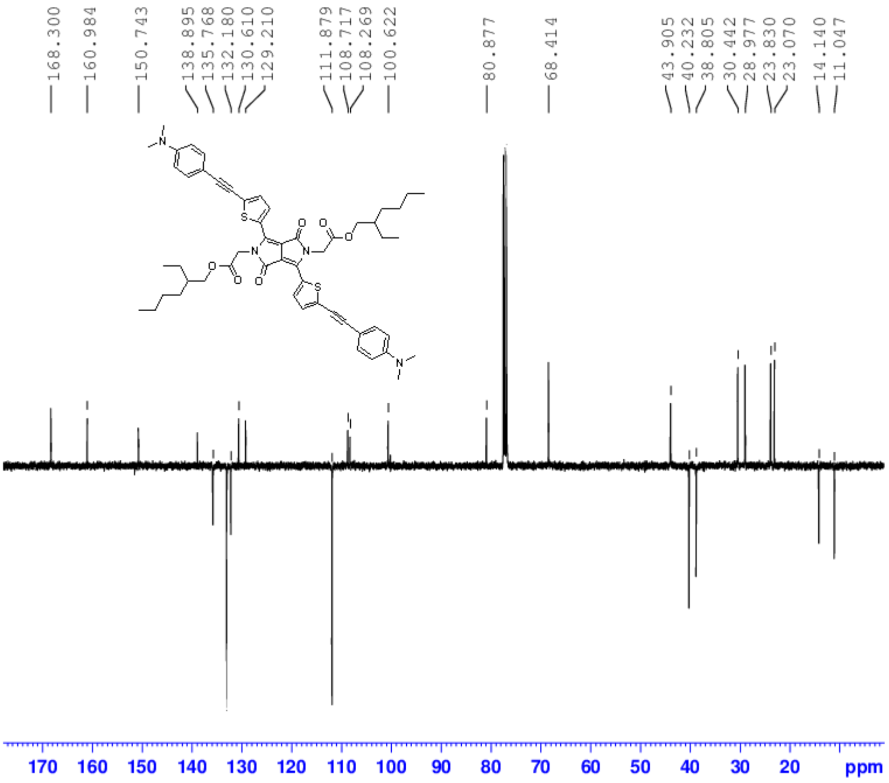

Experimental (up) and calculated (down) MALDI spectra of **1b**

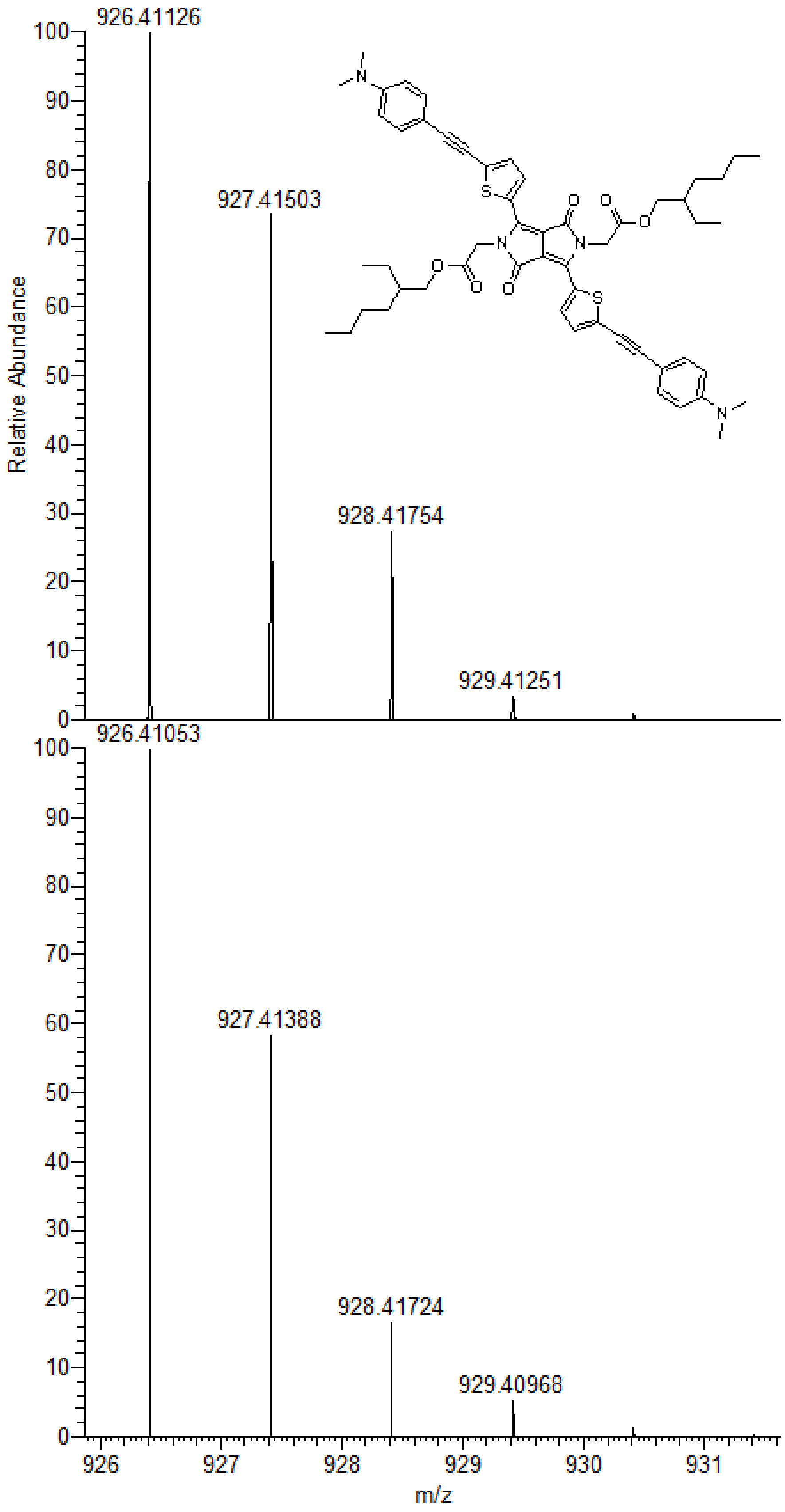

6. ^{1}H and ^{13}C NMR and HR-MALDI-MS spectra of chromophore 2b

^{1}H NMR spectrum (400 MHz, $CDCl_3$, 25 °C) of **2b**

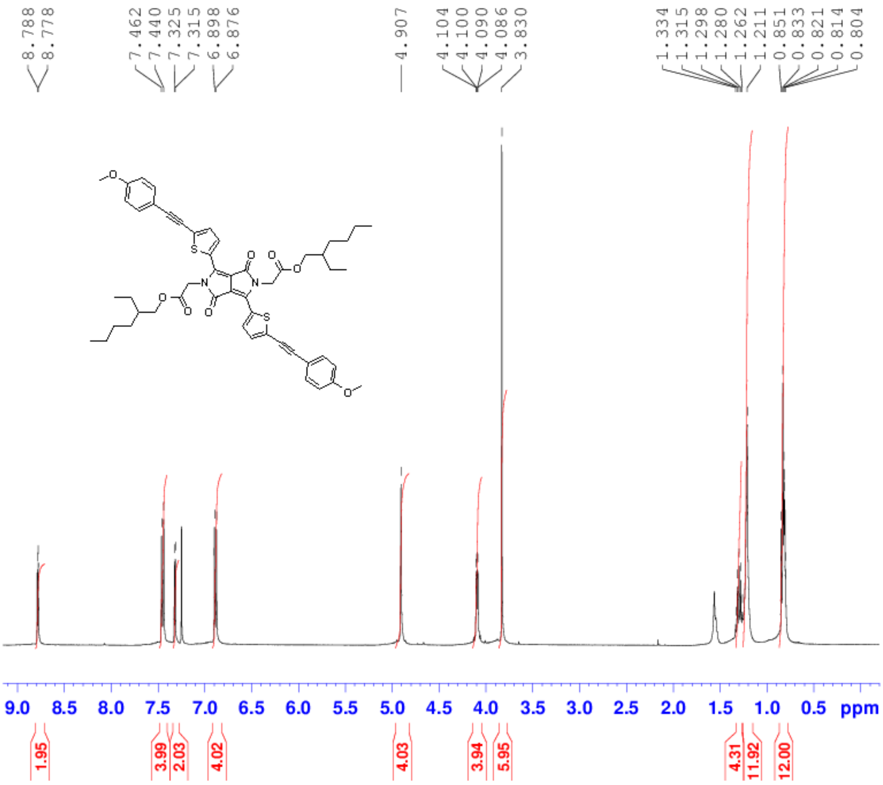

^{13}C NMR spectrum (100 MHz, $CDCl_3$, 25 °C) of **2b**

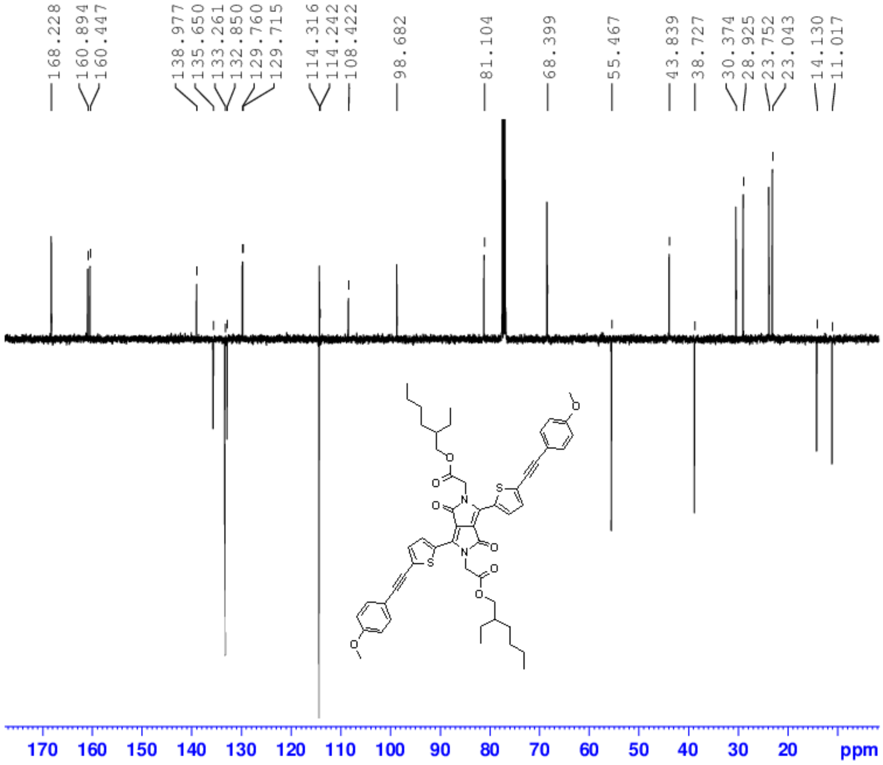

Experimental (up) and calculated (down) MALDI spectra of **2b**

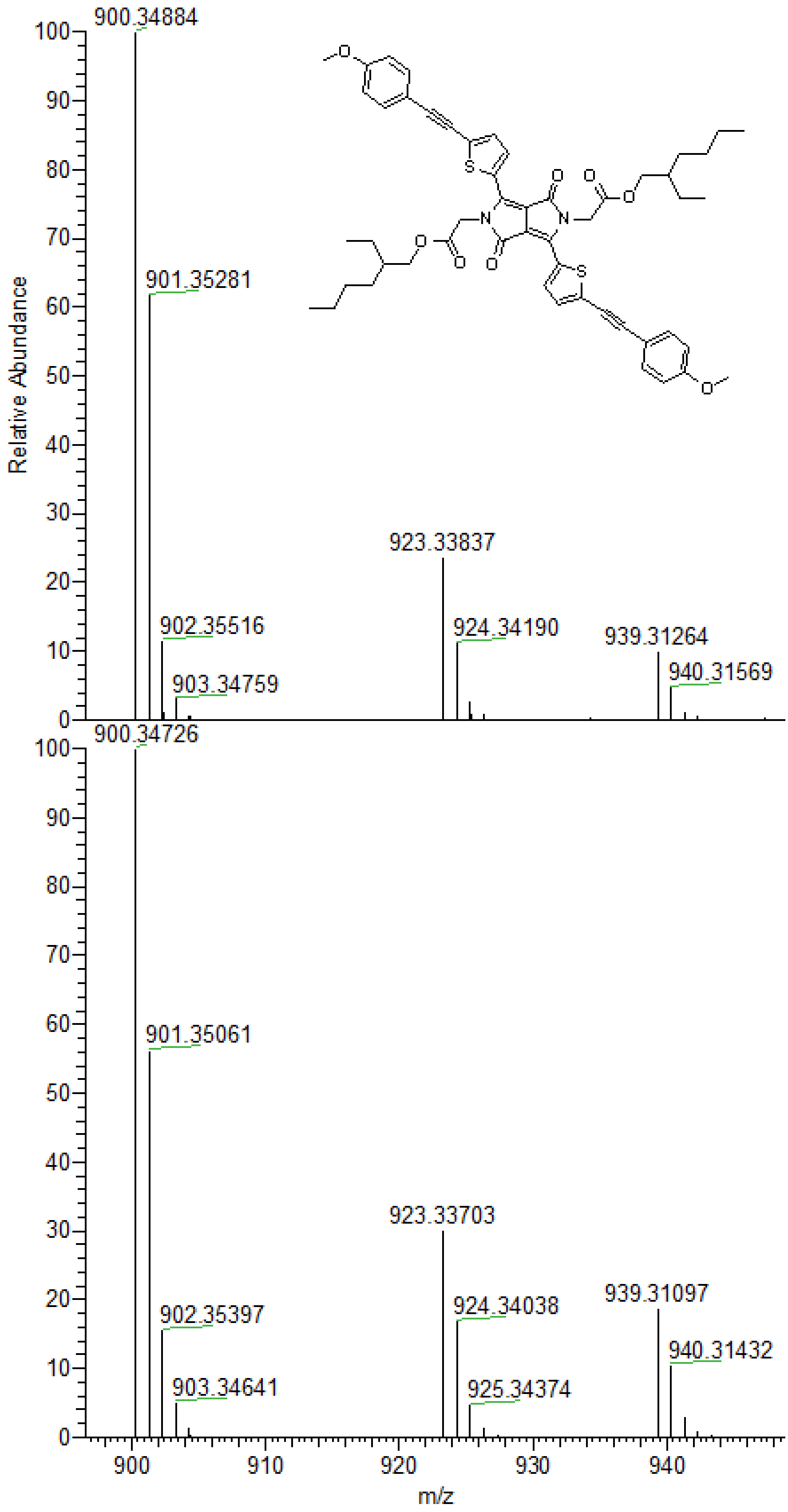

7. ^{1}H and ^{13}C NMR and HR-MALDI-MS spectra of chromophore 3b

^{1}H NMR spectrum (400 MHz, $CDCl_3$, 25 °C) of **3b**

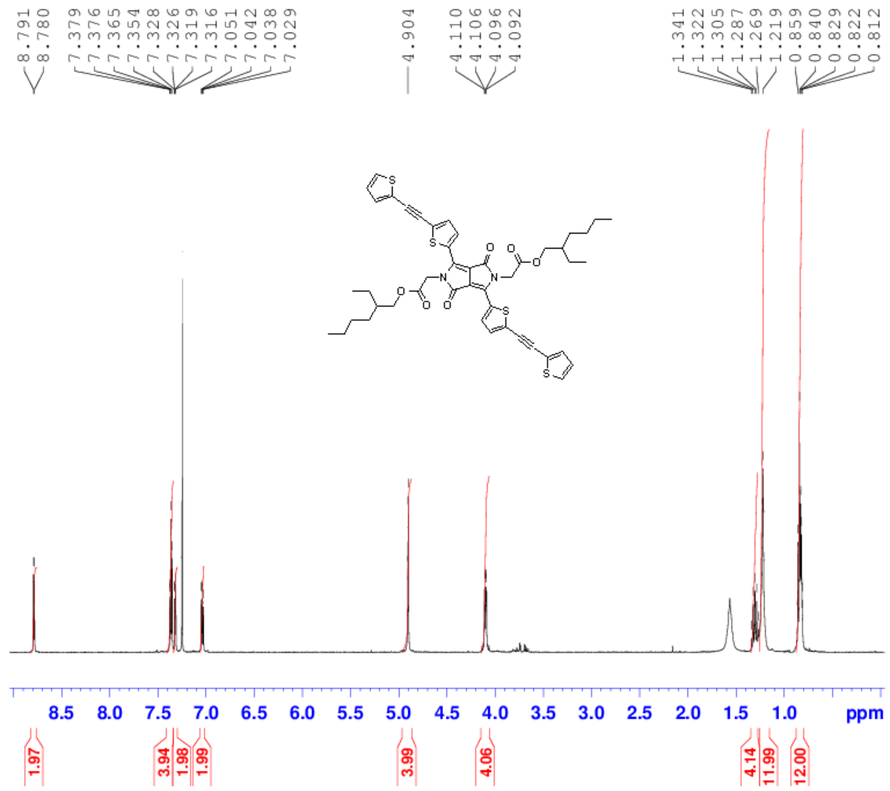

^{13}C NMR spectrum (100 MHz, $CDCl_3$, 25 °C) of **3b**

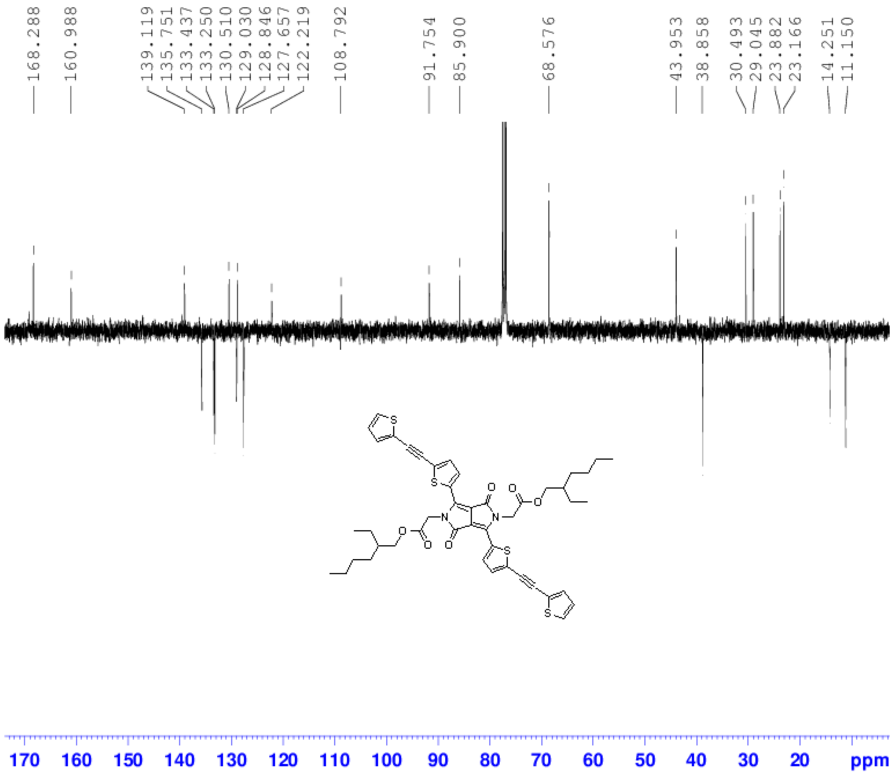

Experimental (up) and calculated (down) MALDI spectra of **3b**

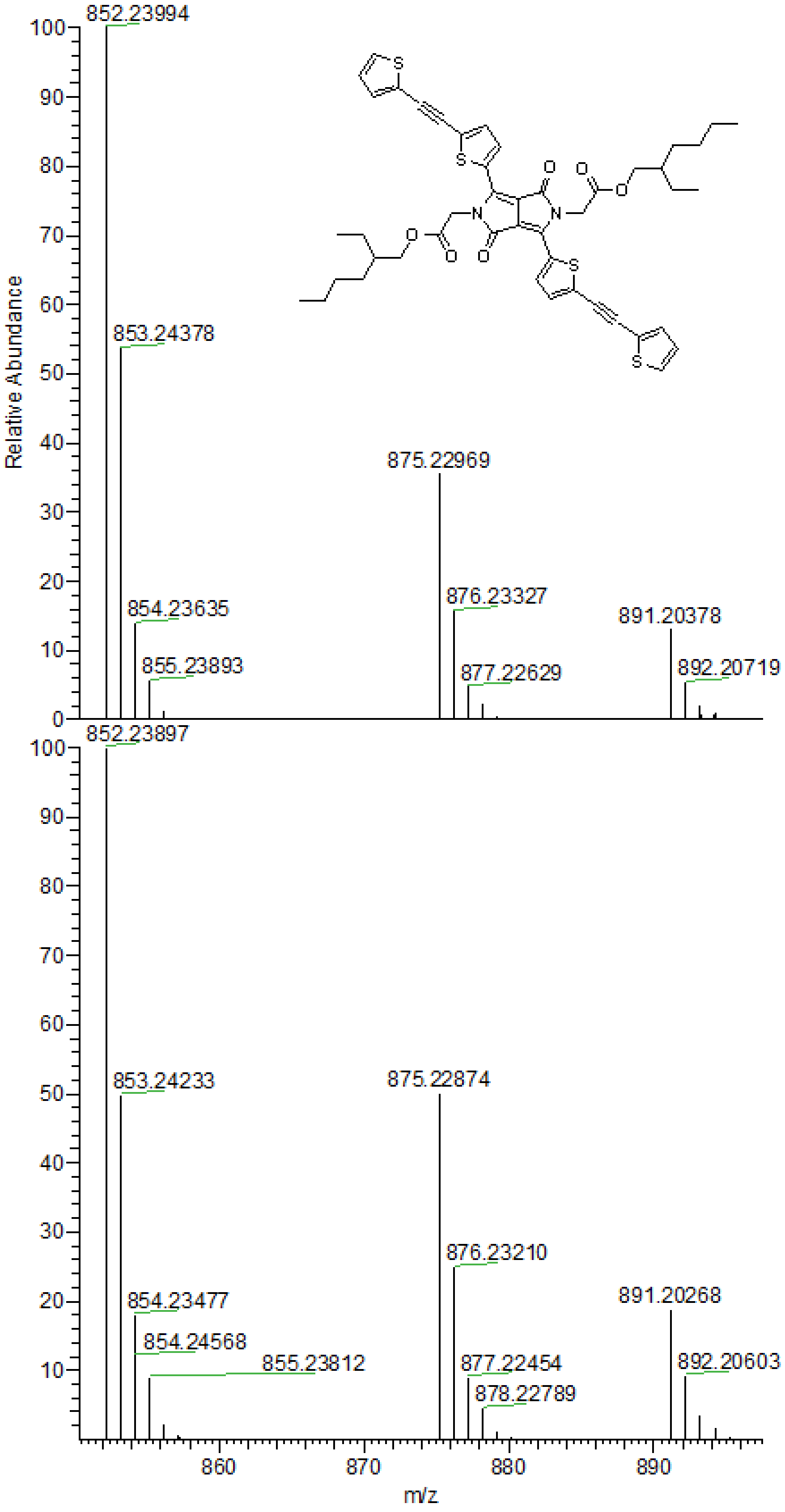

8. ^{1}H and ^{13}C NMR and HR-MALDI-MS spectra of chromophore 4b

^{1}H NMR spectrum (400 MHz, $CDCl_3$, 25 °C) of **4b**

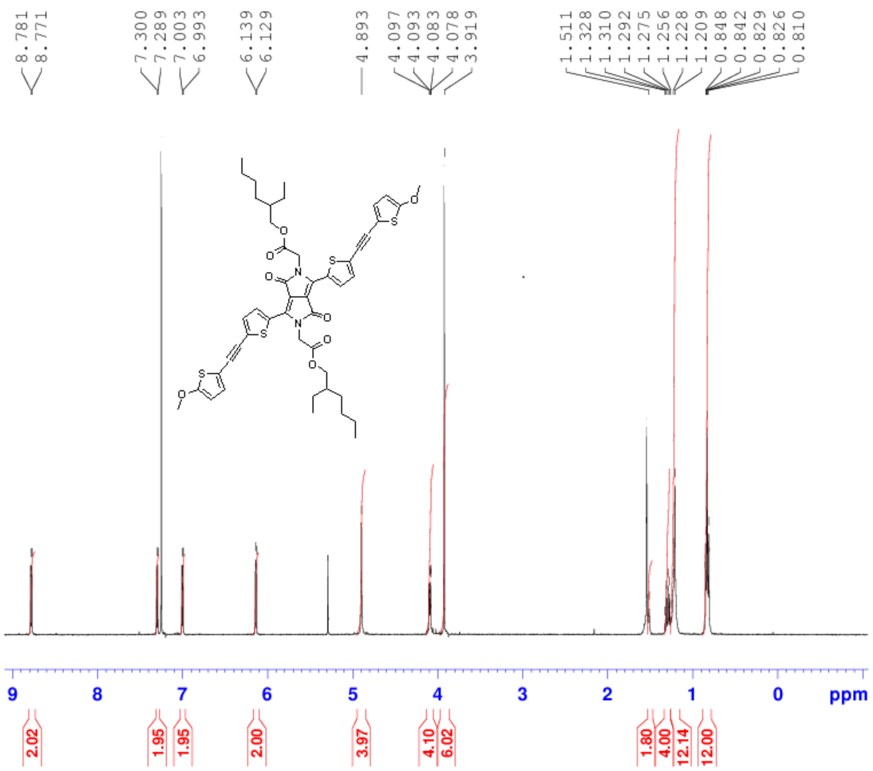

^{13}C NMR spectrum (100 MHz, $CDCl_3$, 25 °C) of **4b**

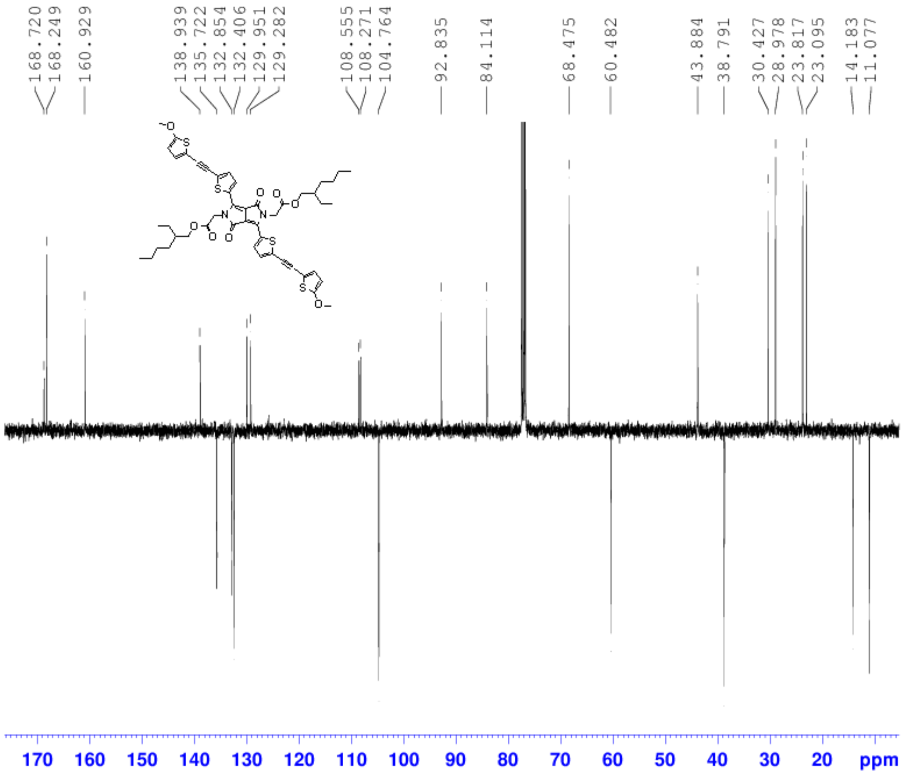

Experimental (up) and calculated (down) MALDI spectra of **4b**

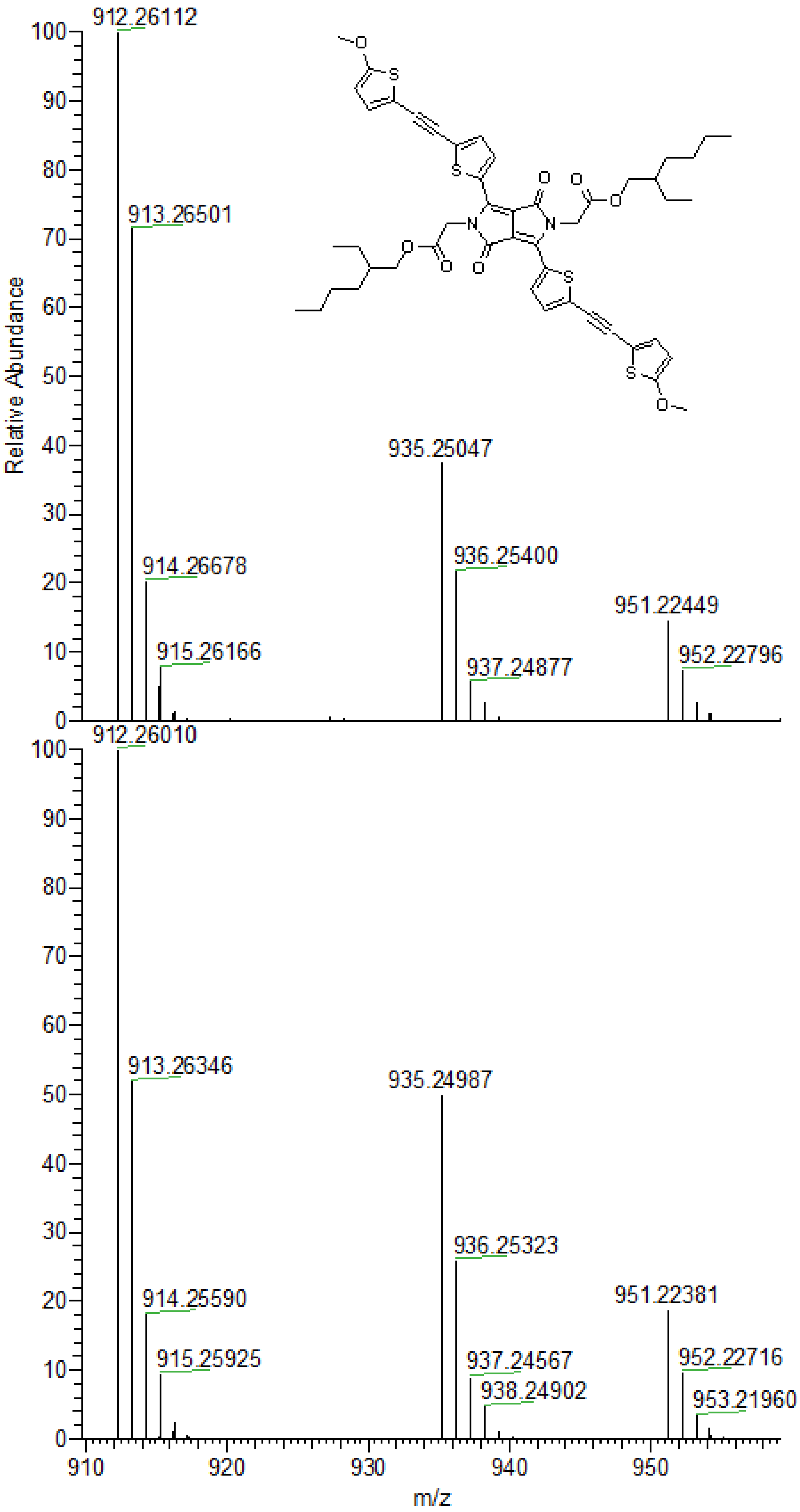

9. ^{1}H and ^{13}C NMR and HR-MALDI-MS spectra of chromophore 5b

^{1}H NMR spectrum (400 MHz, $CDCl_3$, 25 °C) of **5b**

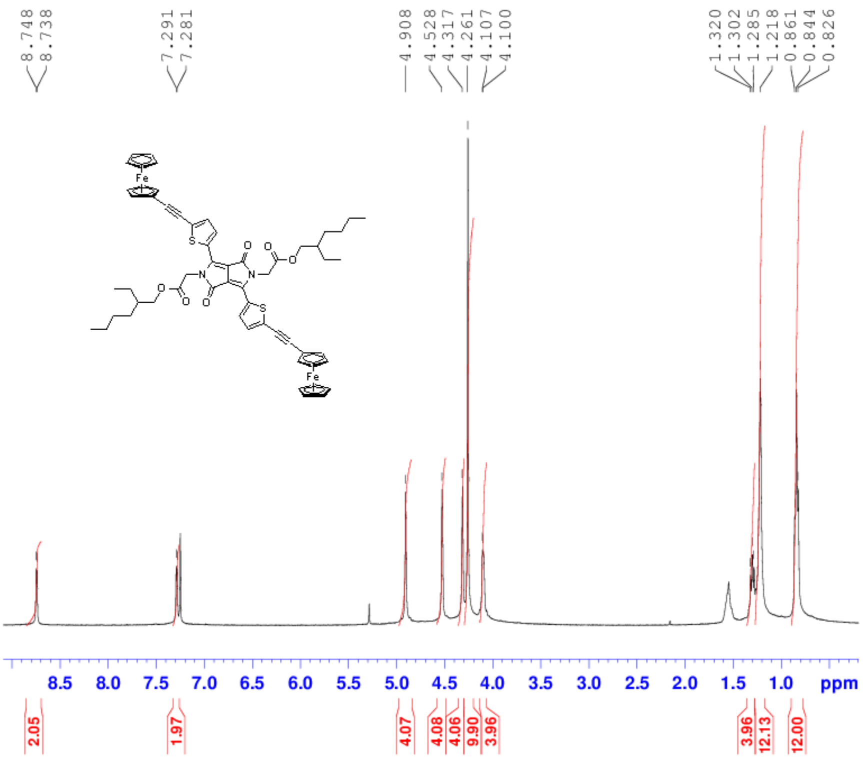

^{13}C NMR spectrum (100 MHz, $CDCl_3$, 25 °C) of **5b**

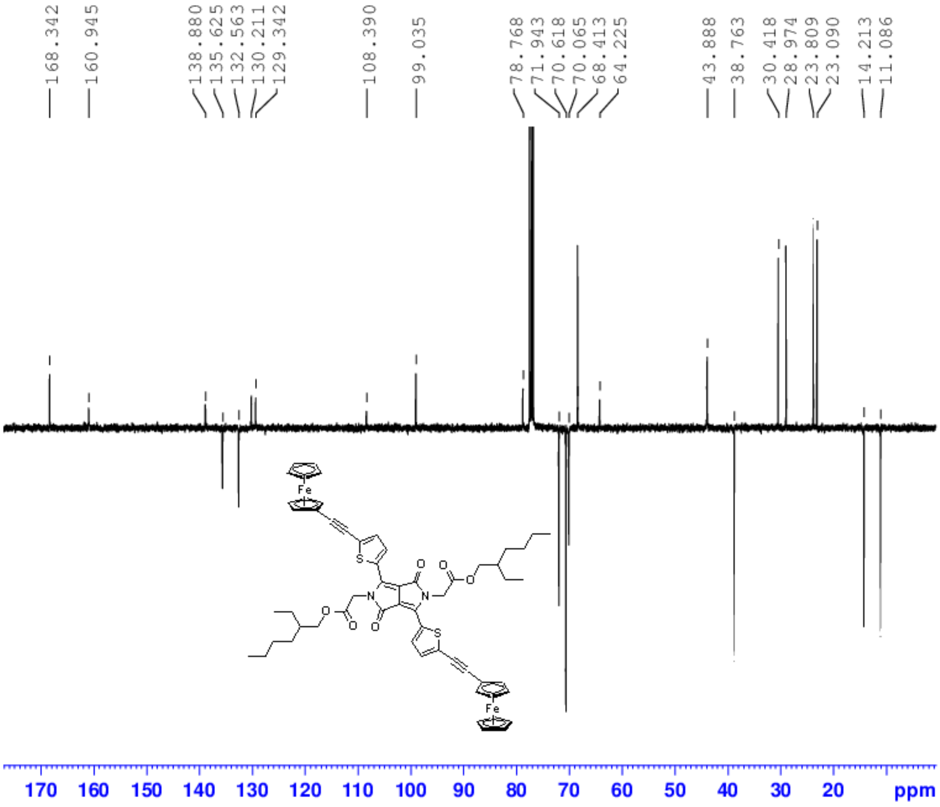

Experimental (up) and calculated (down) MALDI spectra of **5b**

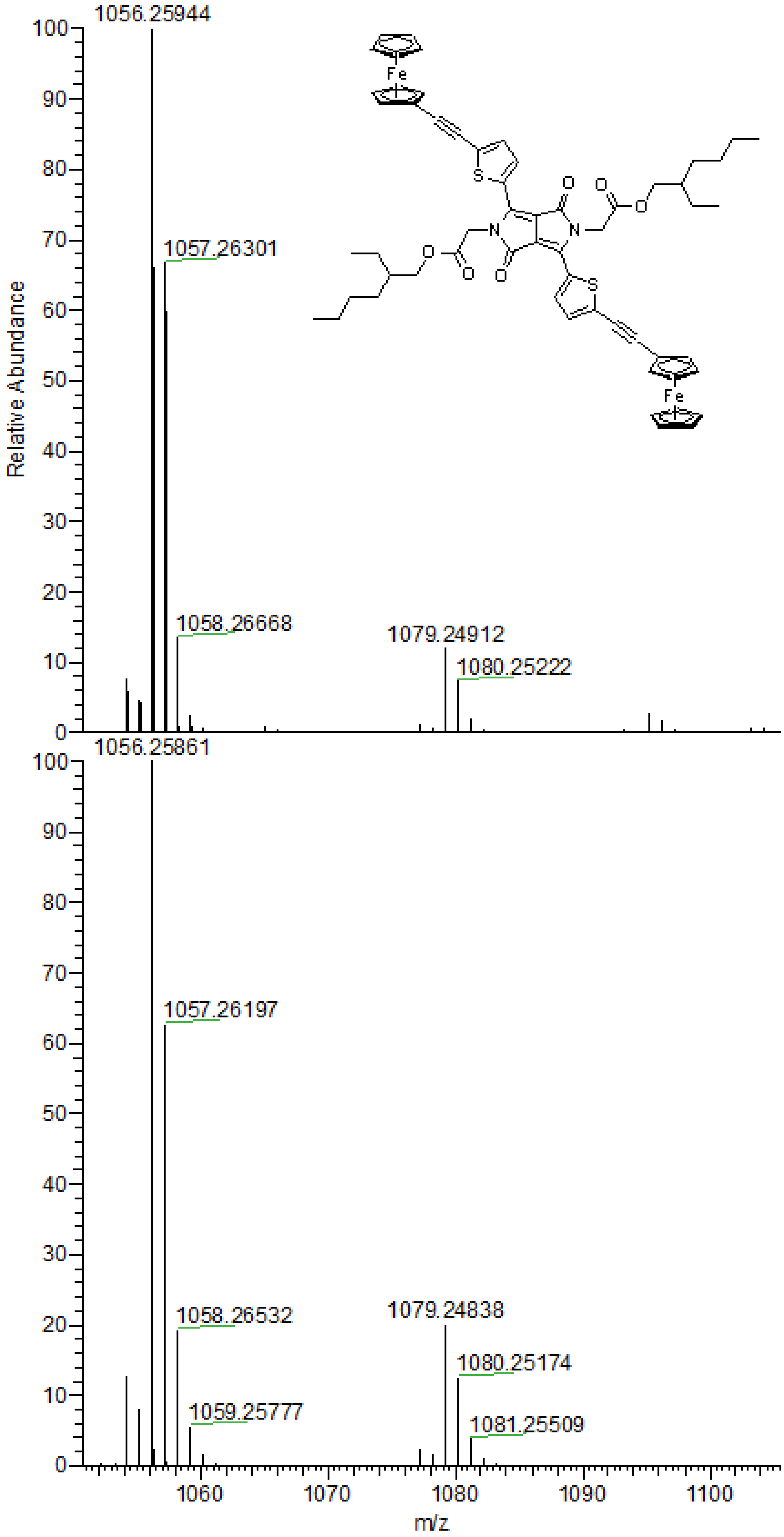

10. Electrochemistry

Representative CV curve of the oxidation and reduction of compound **1a** at Pt electrode in *N*,*N*,-dimethylformamide containing 0.1 M Bu_4NPF_6; scan rate 100mV/s

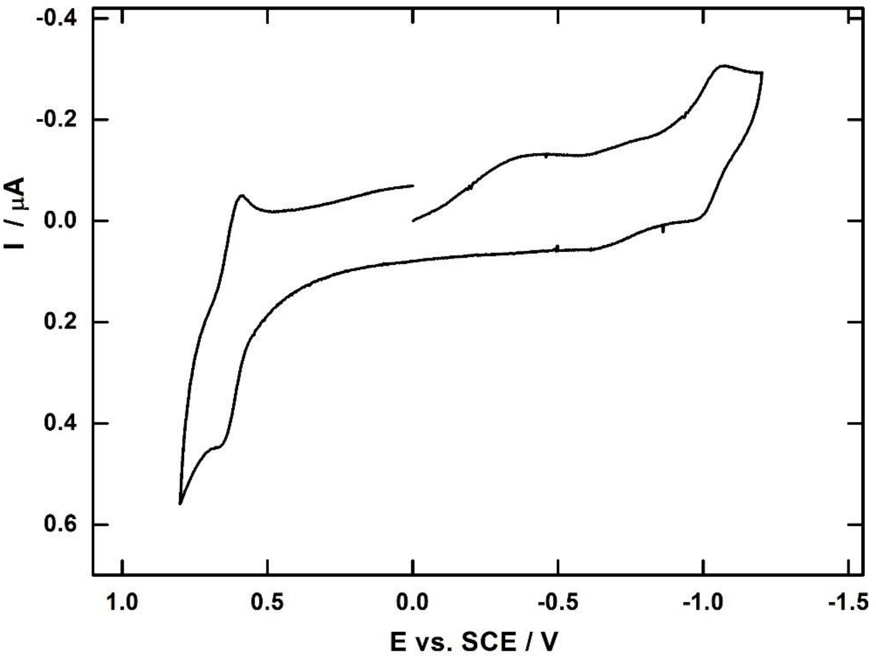

Representative CV curve of the oxidation and reduction of compound **2b** at Pt electrode in *N*,*N*,-dimethylformamide containing 0.1 M Bu_4NPF_6; scan rate 100mV/s

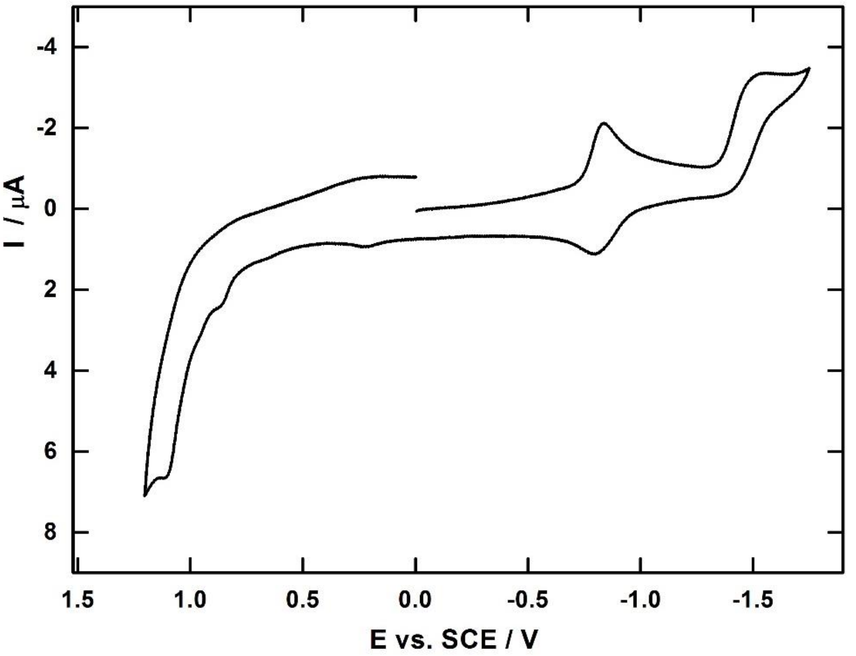

Representative CV curve of the oxidation and reduction of compound **3a** at Pt electrode in *N*,*N*,-dimethylformamide containing 0.1 M Bu_4NPF_6; scan rate 100mV/s

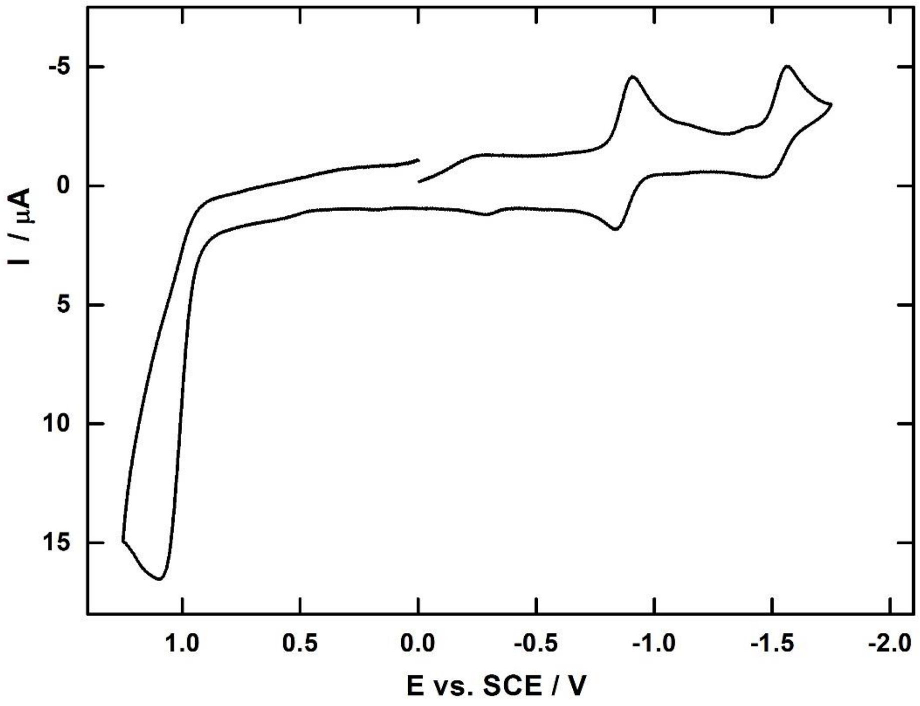

Representative CV curve of the oxidation and reduction of compound **4a** at Pt electrode in *N*,*N*,-dimethylformamide containing 0.1 M Bu_4NPF_6; scan rate 100mV/s

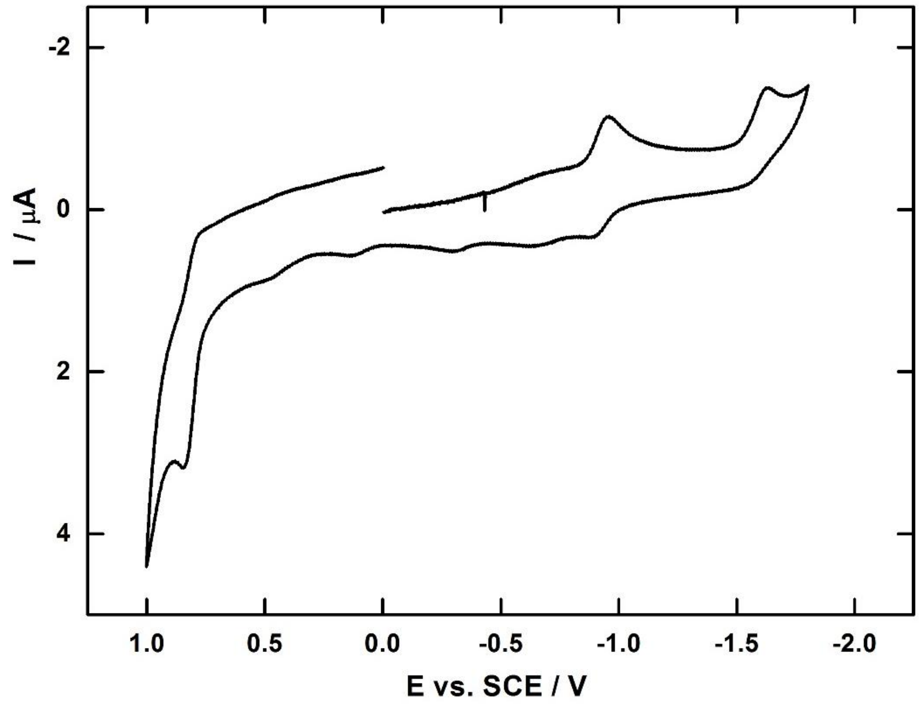

Representative CV curve of the oxidation and reduction of compound **5b** at Pt electrode in *N*,*N*,-dimethylformamide containing 0.1 M Bu_4NPF_6; scan rate 100mV/s

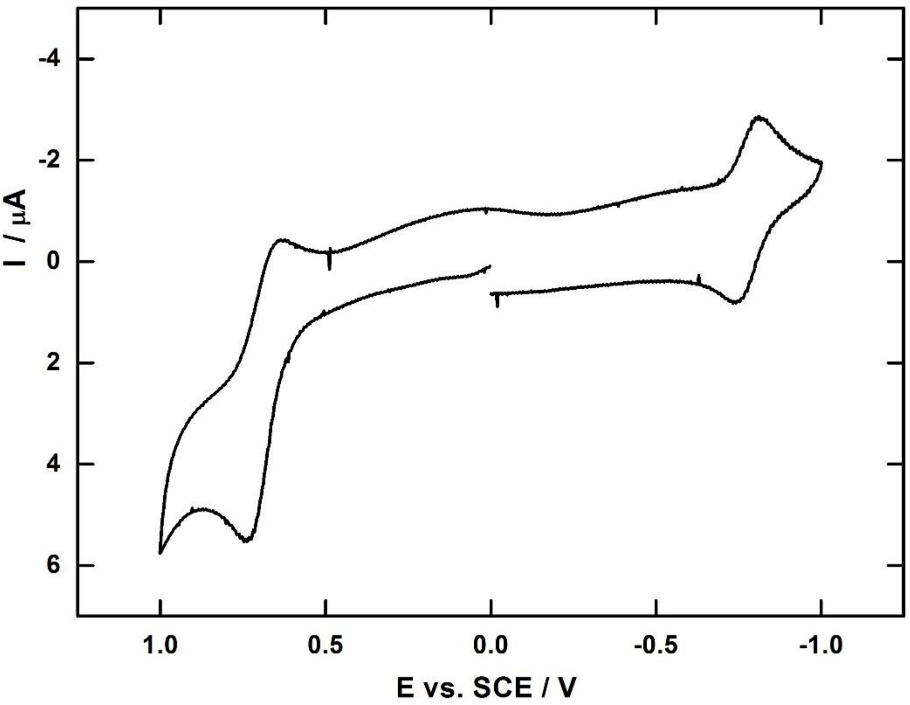

11. Electronic absorption and emission spectra

UV/VIS absorption spectra of chromophores **1a**-**4a** in 1,4-dioxane at concentration 1 × 10^{-5} M

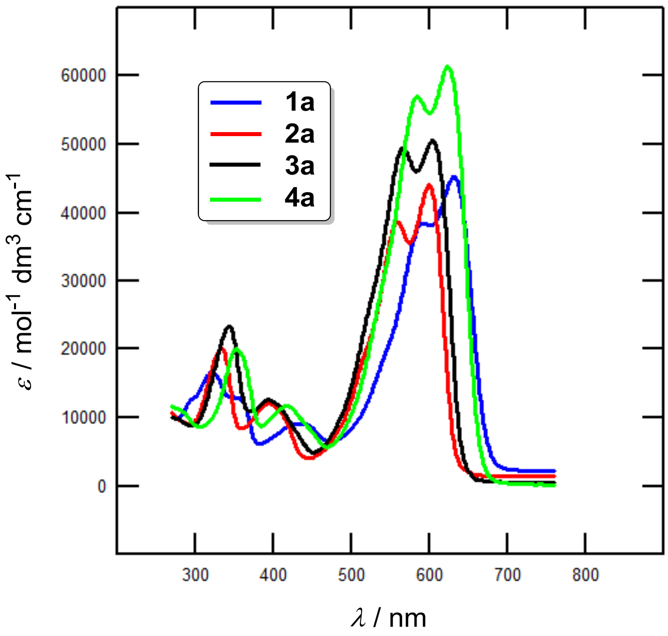

UV/VIS absorption spectra of chromophores **1b**-**5b** in 1,4-dioxane at concentration 1 × 10^{-5} M

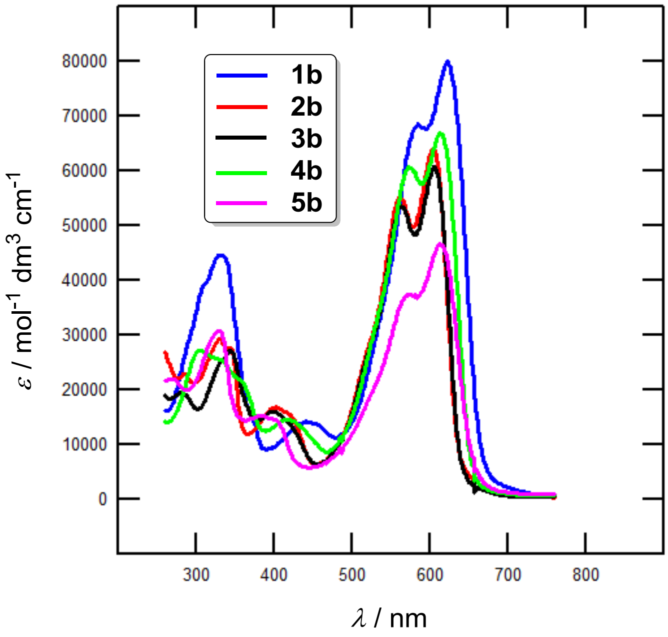

Emission fluorescent spectra of chromophores **1a**-**4a** in 1,4-dioxane

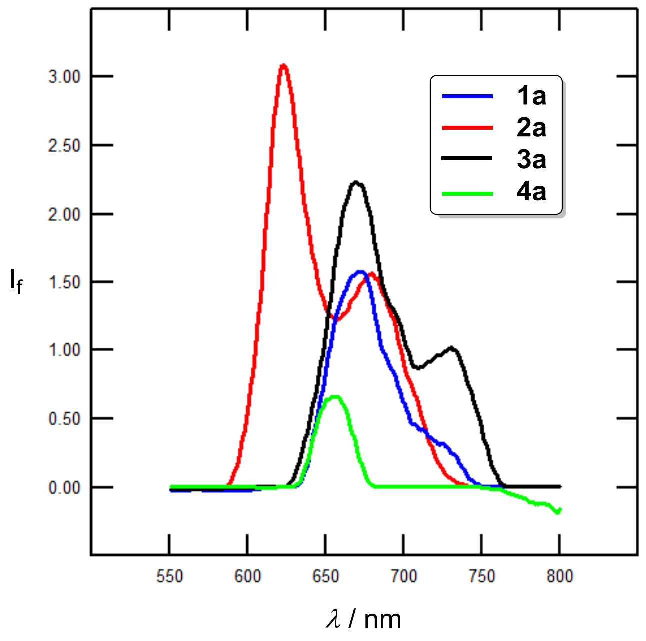

Emission fluorescent spectra of chromophores **1b**-**4b** in 1,4-dioxane

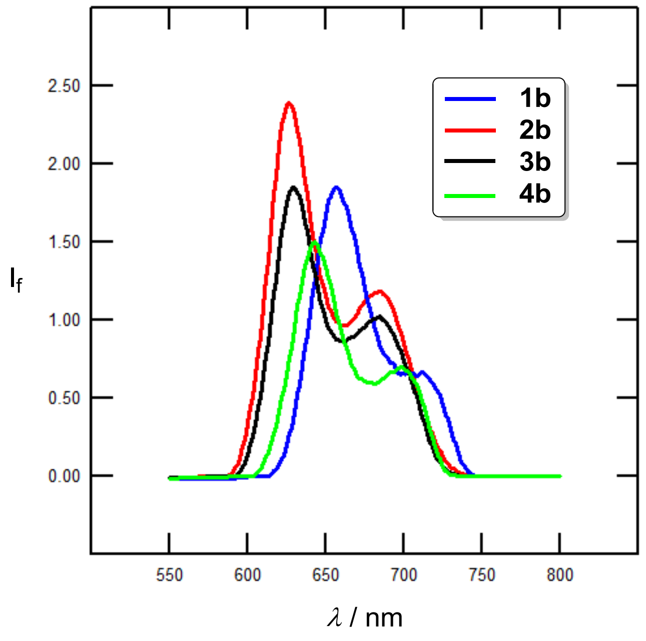

12. HOMO and LUMO visualization

HOMO (red) and LUMO (blue) localizations in **2a**

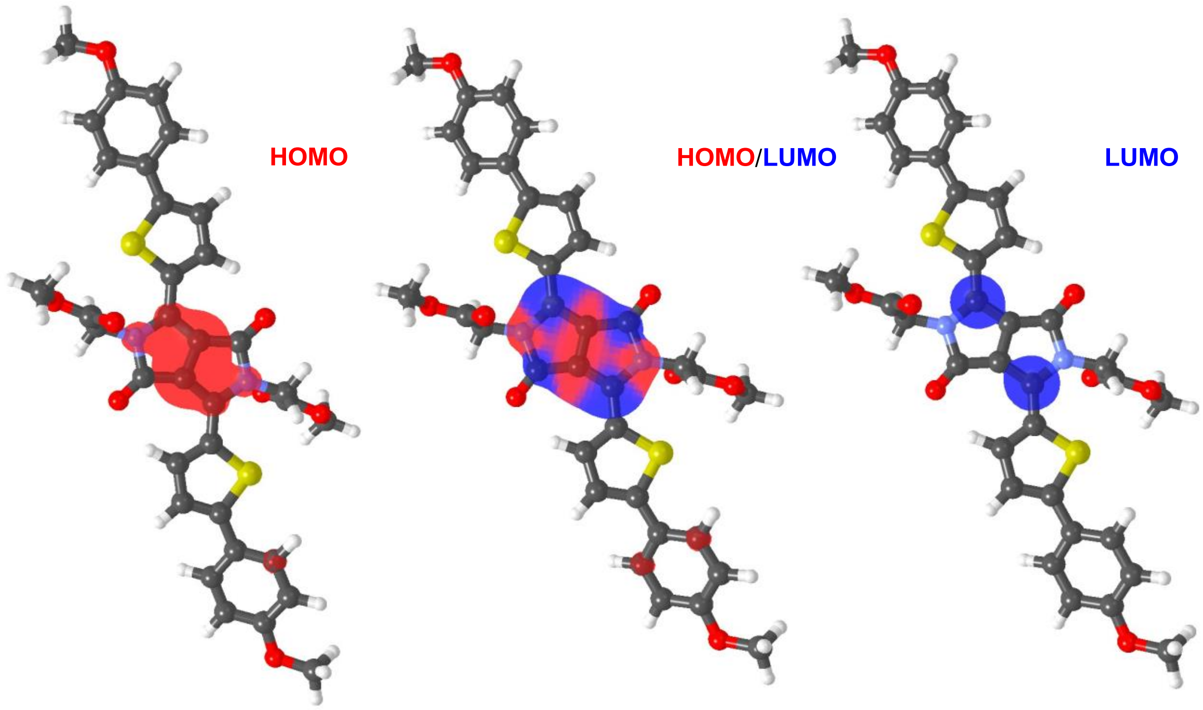

HOMO (red) and LUMO (blue) localizations in **3a**

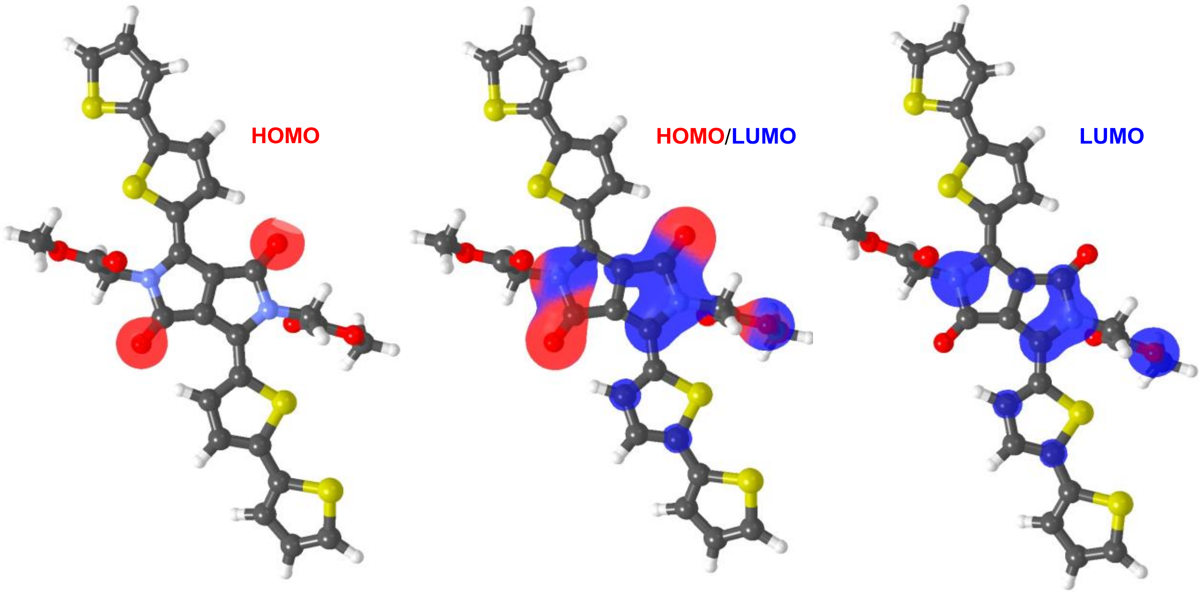

HOMO (red) and LUMO (blue) localizations in **4a**

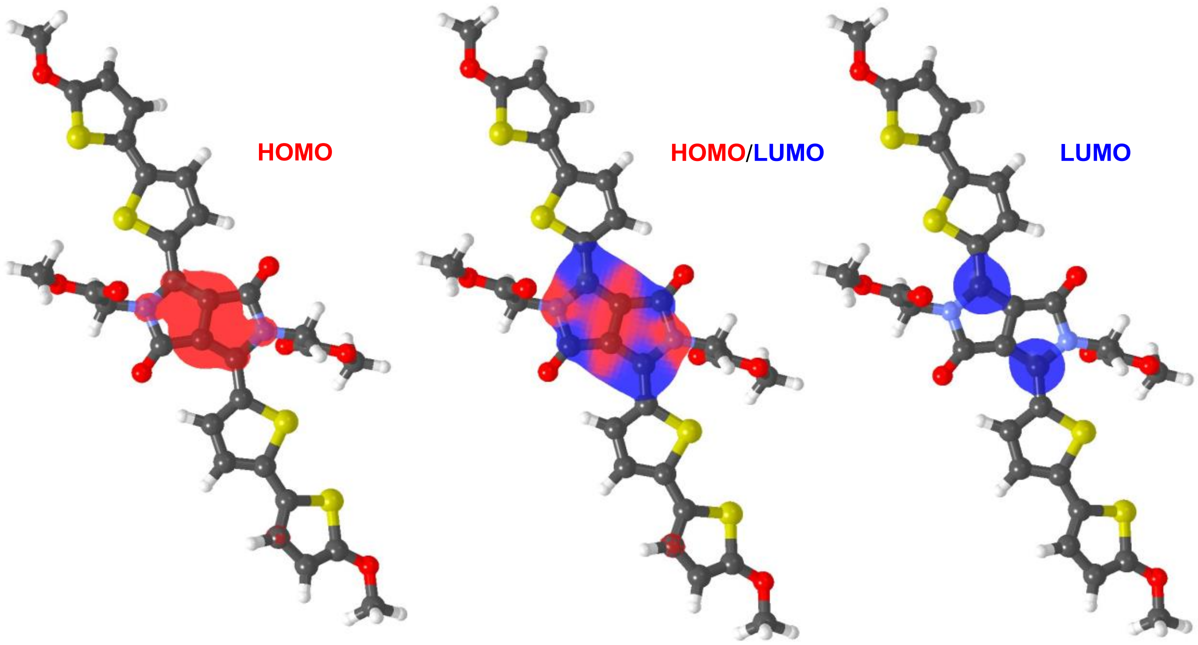

HOMO (red) and LUMO (blue) localizations in **1b**

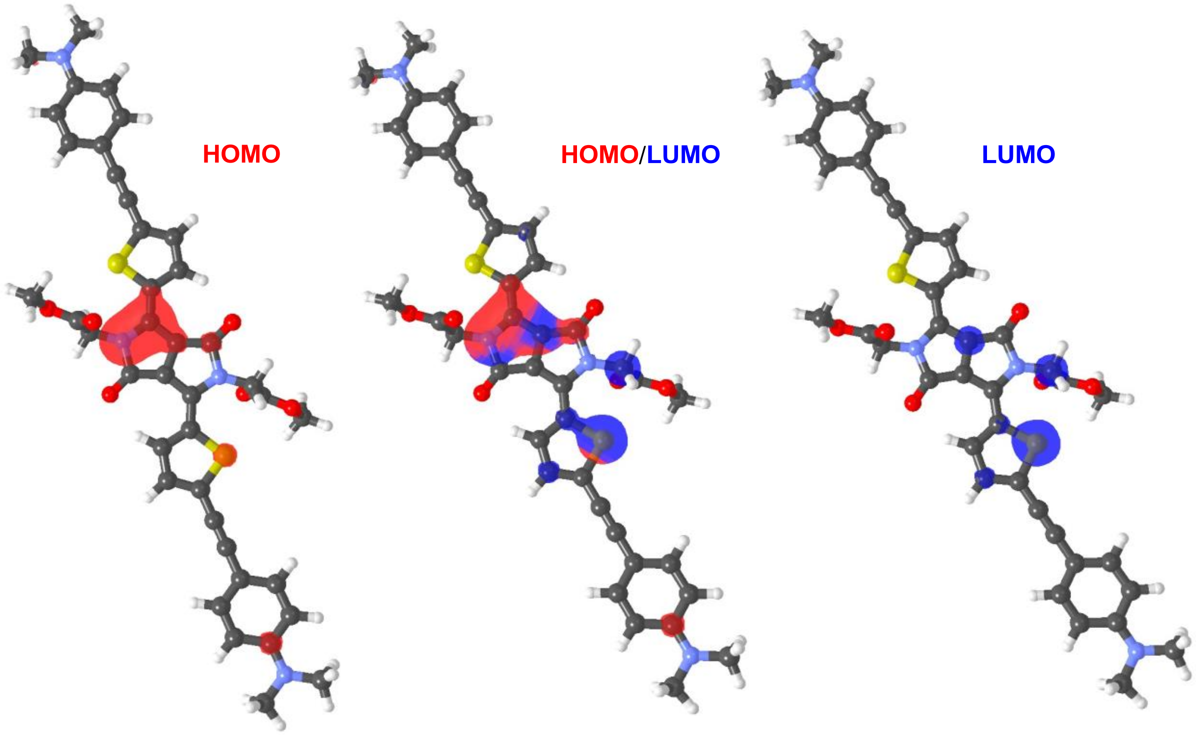

HOMO (red) and LUMO (blue) localizations in **2b**

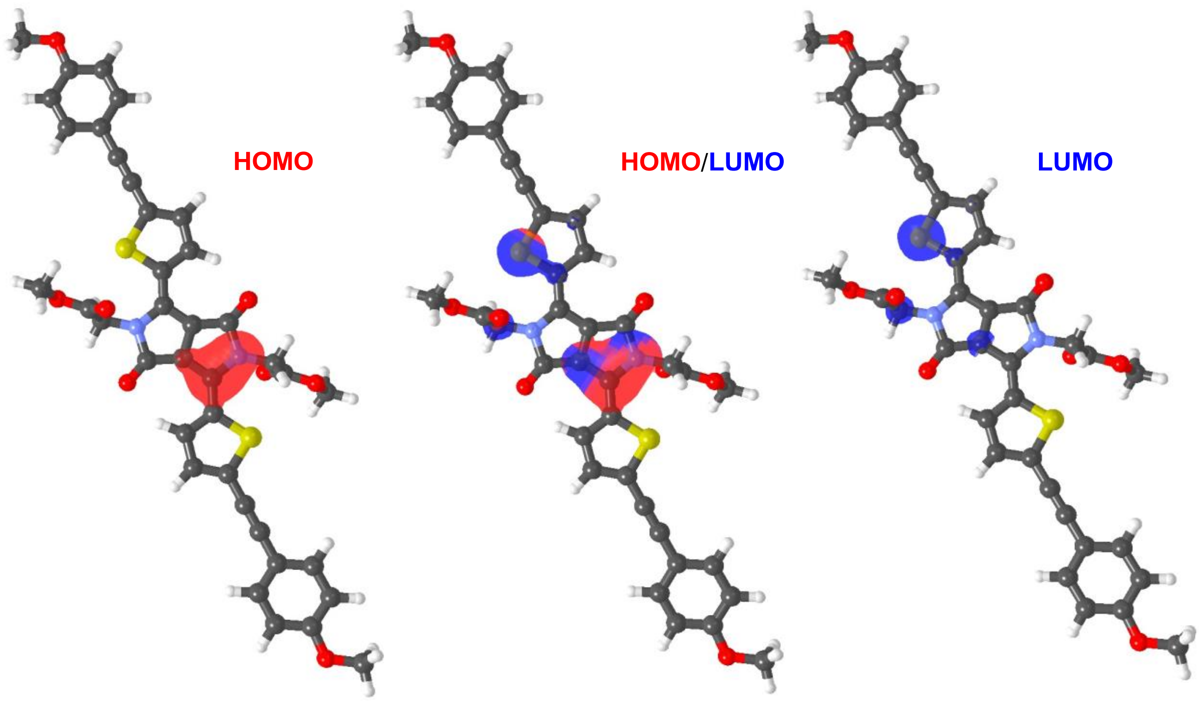

HOMO (red) and LUMO (blue) localizations in **3b**

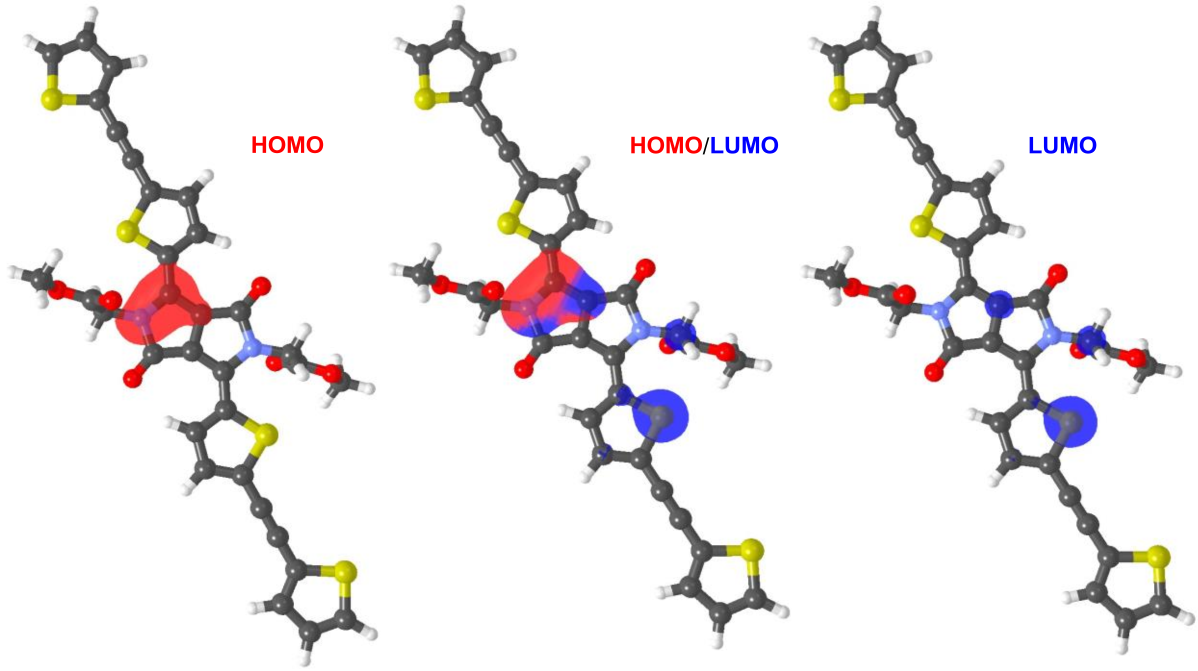

HOMO (red) and LUMO (blue) localizations in **4b**

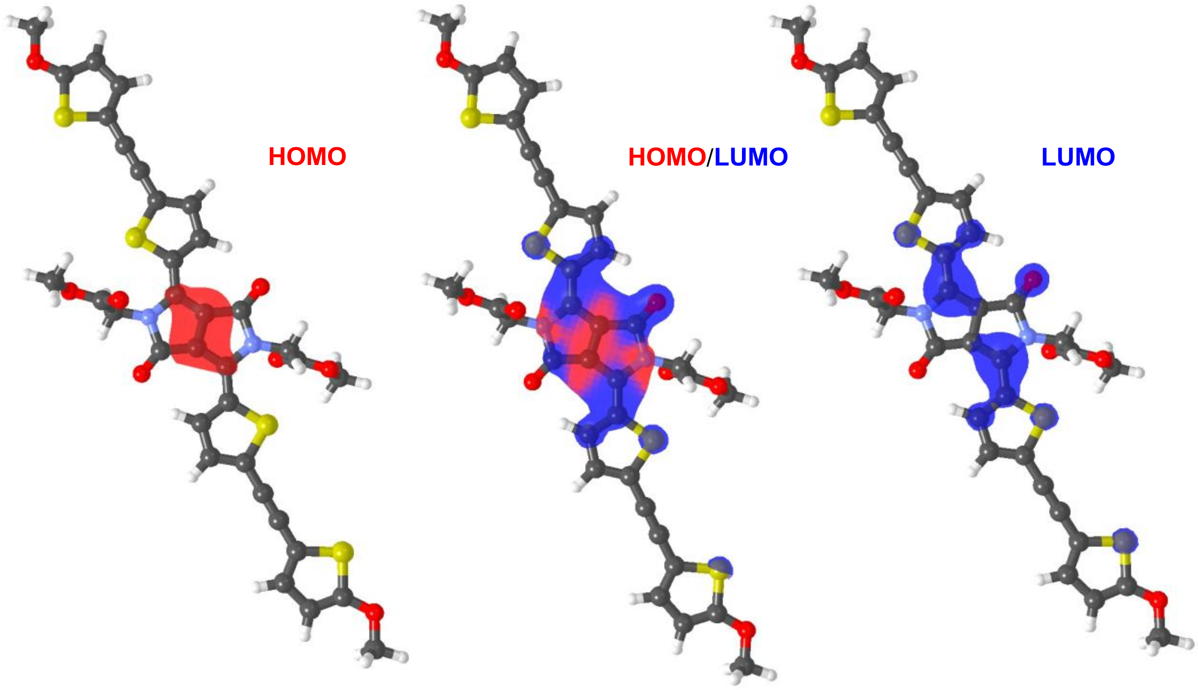

HOMO (red) and LUMO (blue) localizations in **5b**

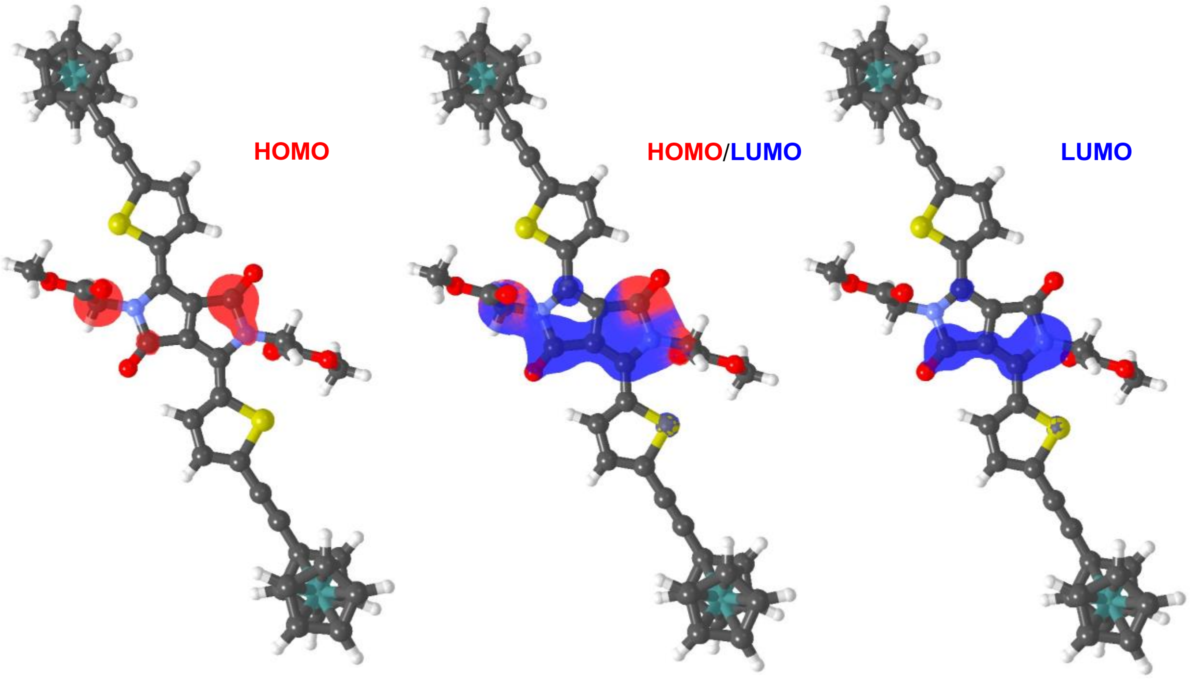

Supplement: File 2 — 1H and 13C NMR spectra, HR-MALDI-MS spectra, CV curves, UV–vis absorption/emission spectra, and HOMO/LUMO localizations. [file Beilstein_J_Org_Chem-13-2374-s002.pdf]
